# Intranasally administered whole virion inactivated vaccine against clade 2.3.4.4b H5N1 influenza virus with optimized antigen and increased cross-protection

**DOI:** 10.1186/s12985-025-02760-4

**Published:** 2025-05-05

**Authors:** Jin-Ha Song, Seung-Eun Son, Ho-Won Kim, Seung-Ji Kim, Se-Hee An, Chung-Young Lee, Hyuk-Joon Kwon, Kang-Seuk Choi

**Affiliations:** 1 Laboratory of Avian Diseases, College of Veterinary Medicine, Seoul National University, 1, Gwanak-ro, Seoul, 88026 Republic of Korea; 2 Avian Influenza Research & Diagnostic Division, Animal and Plant Quarantine Agency, Gimcheon-si, Republic of Korea; 3 Department of Microbiology, College of Medicine, Kyungpook National University, Daegu, Republic of Korea; 4 Research Institute for Veterinary Science, College of Veterinary Medicine, Seoul National University, Seoul, Republic of Korea; 5 Laboratory of Poultry Medicine, Department of Farm Animal Medicine, College of Veterinary Medicine, BK21 PLUS for Veterinary Science, Seoul National University, 1, Gwanak-ro, Seoul, 88026 Republic of Korea; 6 GeNiner Inc, Seoul, Republic of Korea

**Keywords:** Highly pathogenic avian influenza virus, Heat stability, Whole inactivated virus vaccine, Binary ethylenimine, Intranasal administration, Cross-protection

## Abstract

The global spread, frequent antigenic changes, and pandemic potential of clade 2.3.4.4b highly pathogenic avian influenza H5N1 underscore the urgent need for robust cross-protective vaccines. Here, we developed a clade 2.3.4.4b H5N1 whole inactivated virus (WIV) vaccine strain with improved structural stability, productivity, and safety. By analyzing the evolutionary trends of clade 2.3.4.4b H5N1 viruses, we identified a key mutation (R90K) that increases heat stability while preserving antigenicity. Additionally, the PB2 gene of PR8 was replaced with a prototypical avian PB2 gene to increase replication efficiency in embryonated chicken eggs and reduce replication efficiency in mammalian cells, thereby improving productivity and biosafety. We found that our optimized clade 2.3.4.4b H5N1 vaccine strain (22W_KY), inactivated with binary ethylenimine (BEI), had superior antigen internalization into respiratory epithelial cells compared to those inactivated with formaldehyde or beta-propiolactone. Following intranasal administration to mice, the BEI-inactivated 22W_KY also elicited significantly stronger systemic IgG, mucosal IgA, and T-cell responses, especially in the lungs. Protective efficacy studies revealed that the BEI-inactivated 22W_KY vaccine provided complete protection against heterologous viral challenges and significant protection against heterosubtypic viral challenges, with no weight loss and complete suppression of the viral load in the respiratory tract in 2 of 3 mice. These results indicate that the BEI-inactivated 22W_KY vaccine could serve as a promising candidate for a safe, stable, cost-efficient, and broadly protective intranasal influenza vaccine against zoonotic and pandemic threats.

**Supplementary Information:**

The online version contains supplementary material available at 10.1186/s12985-025-02760-4.

## Background

The H5 highly pathogenic avian influenza virus (HPAIV) was first detected in 1996 at a goose farm in Guangdong, China, as the A/goose/Guangdong/1/96 (Gs/Gd-lineage, H5N1). Since its emergence, the virus has undergone continuous mutation, causing significant damage to the poultry industry worldwide [1, 2]. Clade 2.3.4.4b HPAIVs, particularly clade 2.3.4.4b H5N8, dominated globally in 2016–2017 and 2020–2021 but were largely replaced by clade 2.3.4.4b H5N1 in 2021 [3–5]. Recent reports have indicated that clade 2.3.4.4b H5N1 strains exhibit pathogenicity not only in poultry but also in a wide range of species, including birds of prey [6], carnivores [7–9], and marine mammals [10, 11]. Even mammal-to-mammal transmission of the clade 2.3.4.4b H5N1 strain has been reported in dairy cows and marine mammals [5, 12], and this, coupled with the rapid evolutionary capacity of influenza viruses, highlights the significant pandemic potential of the H5N1 strain, thereby raising serious global attention and concern [13, 14]. Therefore, the development of a clade 2.3.4.4b H5N1 vaccine with high immunogenicity and a broad protective spectrum that can address the frequent mutations of HPAIVs is imperative. Such a vaccine should not only provide robust protection for poultry but also be applicable to mammalian species, addressing the growing zoonotic and pandemic risks associated with this virus.

There are several critical aspects of whole inactivated virus (WIV) strain development for vaccines: efficient antigen production, antigen stability, and safety assurance. The generation of robust protective antibody responses by WIVs typically is dependent on the presence of a high antigenic content. Additionally, achieving high vaccine yields is crucial for economic viability. Efficient propagation of viruses in scalable systems, such as embryonated eggs or cell culture platforms, ensures the production of sufficient antigen quantities to meet global demand while supporting cost-effective manufacturing processes. Another key consideration in WIV vaccine development is the stability of the virus. Structurally stable vaccine viruses are less sensitive to temperature fluctuations during storage and transportation, ensuring that vaccine efficacy is maintained even outside refrigeration or freezing conditions. This decreased sensitivity of temperature fluctuations significantly increases vaccine virus stability, extending its effective shelf life and usability. Reverse genetics are traditionally used in the design of WIV vaccines for influenza A virus, with the hemagglutinin (HA) and neuraminidase (NA) genes from circulating strains being combined with the six internal genes from the A/Puerto Rico/8/1934 (PR8, H1N1) virus, which is well characterized for vaccine production [15]. However, the use of PR8 has certain limitations, including suboptimal replication efficiency in embryonated eggs for specific strains and the presence of multiple mutations associated with mammalian pathogenicity (e.g., E627K) [16, 17]. In our previous study, we demonstrated that replacing the PB2 gene of PR8 with the PB2 gene from A/Chicken/01310/Korea/01310/2001 (01310, H9N2) significantly suppressed virus replication efficiency in mammalian cells and mice [16, 18]. Furthermore, we found that the PB2 gene of 01310, modified with I66M, I109V, and I133V mutations (referred to as 310-MVV), increased the replication efficiency of Y280-lineage H9N2 viruses compared with the wild-type PB2 gene of 01310 [19].

The initial step for influenza viruses to infect host cells involves targeting epithelial cells in both the upper and lower respiratory tracts. Establishing local immunity at the respiratory mucosal level is therefore critical for preventing respiratory viral infections [20]. Most WIV vaccines against influenza viruses are administered intramuscularly, focusing primarily on systemic humoral immune responses that generate neutralizing antibodies that target HA and NA. However, these WIV vaccines induce minimal local immune responses in the respiratory mucosa and fail to elicit robust cellular immunity [21]. This limitation results in protection that is often restricted to specific virus strains. In contrast, mucosal vaccines are considered promising because they induce high levels of secretory immunoglobulin A (sIgA) and cellular immunity in the respiratory mucosa [22–24]. This ability enables protection against primary infection in both the upper and lower respiratory tract and offers cross-protection against diverse virus strains [25, 26]. Currently, the only approved influenza mucosal vaccine is a live attenuated influenza virus (LAIV) vaccine, which can strongly stimulate nasal and cellular immunity [23, 27]. However, the potential for virus mutation and reassortment raises safety concerns, limiting the application of this LAIV vaccine. Several efforts have been made to utilize WIV vaccines as mucosal vaccines, as the risks associated with LAIV vaccines are eliminated with WIV vaccines [28–30]. However, significant challenges are faced with WIV vaccines in terms of overcoming the physical barriers of the mucosal environment, such as mucus, cilia, and tight epithelial junctions, leading to limited efficacy [24]. For an effective WIV mucosal vaccine, antigens must efficiently penetrate the mucosal barrier and be absorbed by respiratory epithelial cells. Additionally, the vaccine must exhibit high stability and retention within the nasal mucosa to withstand the local environment and for its efficacy to be maintained [31]. The development of a stable and robust WIV strain optimized for mucosal delivery is thus essential for advancing WIV-based mucosal vaccine strategies.

In this study, we aimed to optimize a clade 2.3.4.4b H5N1 WIV vaccine strain by specifically improving antigen production efficiency, structural stability, and biosafety and to develop a WIV-based intranasal vaccine capable of inducing cross-protective immunity. We conducted a phylogenetic analysis of clade 2.3.4.4b H5N1 viruses, identified a novel mutation (R90K) that could increase the structural stability of the WIV strain, and incorporated this mutation into our vaccine. Additionally, we employed bioengineering by modifying the PB2 gene to increase viral replication efficiency in embryonated chicken eggs (ECEs) while simultaneously reducing viral fitness in mammalian cells. Finally, to formulate an effective WIV-based intranasal vaccine, we compared the effects of three different inactivation reagents—formaldehyde (F/A), β-propiolactone (BPL), and binary ethylenimine (BEI)—on vaccine efficacy by assessing humoral and cellular immune responses and evaluating protective efficacy against heterologous and heterosubtypic viral challenge in a mouse model.

## Materials & methods

### Viruses, eggs, and cells

The viruses used in this study, A/Puerto Rico/8/1934 (PR8, H1N1) and A/Wild duck/Korea/SNU50-5/2009 (SNU50-5, H5N1) were propagated in ten-day-old specific-pathogen-free (SPF) embryonated chicken eggs (ECEs) (VALO Biomedia GmbH) and stored in aliquots at -80 °C until use. Viral titers were determined and expressed as either 50% Egg Infective Dose (EID_50_) or Tissue Culture Infective Dose (TCID_50_) in Madin-Darby canine kidney (MDCK) cell. The 293T (Korea Cell Line Bank, KCLB, 21573) and MDCK (Korean Collection for Type Cultures, KCTC, AC30015) cell lines were cultured in Dulbecco’s Modified Eagle Medium (DMEM, Gibco, MA, USA) supplemented with 10% Fecal Bovine Serum (FBS, Gibco) and penicillin-streptomycin (Pen-Strep, Gibco). The A549 (Korean Cell Line Bank, KCLB, 10185) cell lines were cultured in DMEM F-12 (Gibco) supplemented with 10% FBS and Pen-Strep. The Calu-3 (Korea Cell Line Bank, KCLB, 30055) cell line was maintained in minimum essential medium with Glutamax (MEM + Glutamax, Gibco), supplemented with 10% FBS, Pen-Strep, 1% HEPES (Gibco), 1% non-essential amino acids (NEAA, Gibco), and 1% sodium pyruvate (Gibco). All cell lines were incubated at 37 °C in a 5% CO_2_ environment.

### Phylogenetic analysis

Genetic and phylogenetic analysis was conducted on the clade 2.3.4.4b H5N1 hemagglutinin amino acid sequences isolated in East Asia. A total of 79 sequences collected from East Asia between October 2021 and February 2023 were obtained from the Global Initiative for Sharing All Influenza Data (GISAID) EpiFlu database [32]. These sequences were aligned using the MAFFT version 7 online tool (https://mafft.cbrc.jp/alignment/server/index.html). To examine evolutionary trends over time, Bayesian Evolutionary Analysis Sampling Trees (BEAST, v1.10.4) analysis was performed. The General Time Reversible (GTR) + γ substitution model was applied, and an uncorrelated relaxed clock was utilized to account for evolutionary rate variation. The analysis was specified with a coalescent constant size tree prior. Three independent runs of 50 million Markov Chain Monte Carlo (MCMC) chains were conducted, and the runs were combined using Log Combiner v1.10.4. The effective sample size (ESS) per parameter was confirmed to be over 200 using Tracer v1.7.2. The final maximum clade credibility (MCC) tree was annotated with Tree Annotator v1.10.4 and visualized in FigTree v1.4.4. To broaden the analysis beyond East Asia, additional clade 2.3.4.4b H5N1 sequences reported in Europe and North America during the same period were included. From 1,633 European and 1,292 North American sequences available in GISAID, 167 European and 83 North American sequences were randomly selected. These sequences, alongside those from East Asia, were processed using the same alignment and phylogenetic methods to generate an MCC tree.

### Generation Recombinant clade 2.3.4.4b H5N1 vaccine strain by reverse genetics system

The amino acid sequences of HA and NA (*n* = 3004) from clade 2.3.4.4b H5N1, isolated in East Asia, North America, and Europe between October 2021 and February 2023, were aligned using the MAFFT tool to obtain consensus sequences. The hemagglutinin consensus sequence was modified to replace the polybasic cleavage site (RERRRKR) with a monobasic site (ASGR), similar to that of the Korean H9N2 LPAI virus, A/Chicken/Korea/KBNP-0028/2000 (H9N2) [33], and cloned into the pHW2000 vector (22 W HA). The neuraminidase consensus sequence (22 W NA) was also cloned into the pHW2000 vector. The 22 W HA and 22 W NA genes, along with six internal genomic segments (PB2, PB1, PA, NP, M, NS) from PR8 and a PB2 gene from 01310 containing I66M, I109V, and I133V (MVV) mutations [16, 19, 34], were cloned into the pHW2000 vector. Plasmids containing the R90K/A93T/S125N/N193D mutation in the 22 W HA gene (named 21 W HA) and six additional plasmids with individual mutations (R90K, A93T, S125N, N193D, H110Y, R90K/H110Y) in 22 W HA were engineered using the Muta-direct site-directed mutagenesis kit (iNtRON, Seongnam, Republic of Korea).

Recombinant viruses were generated using a pHW2000 plasmid-based reverse genetics system [35]. The 293T cell line was seeded in 6-well plates, and 300 ng of each plasmid mix was transfected into the cells using Lipofectamine 2000 (Invitrogen, MA, USA) following the manufacturer’s instructions. The cells were incubated at 37 °C in a CO_2_ incubator for 24 h. 24 h post-incubation, the cells were supplemented with Opti-MEM (Gibco) and tosyl phenylalanyl chloromethyl ketone (TPCK)-treated trypsin (Sigma-Aldrich, MO, USA). After an additional 24-hour incubation, 200 µl of the cell supernatant was inoculated into ten-day-old SPF eggs and incubated for 3 days at 37 °C. The presence of recombinant viruses was confirmed using a hemagglutination (HA) assay with 1% (v/v) chicken red blood cells (cRBC). Each generated recombinant virus was confirmed by RT-PCR and Sanger sequencing.

### Heat stability analysis

To compare the heat stability of recombinant viruses and inactivated viruses, each virus or inactivated virus was diluted to 32 (2^5^) HAU and subjected to heat treatment at 52 °C for 0.5, 1, 1.5, 2, 3, and 4 h. The HA titers of each sample were measured and recorded.

### Replication efficacy in embryonated chicken eggs (ECEs)

The replication efficiency of each recombinant virus was determined by measuring the 50% egg infectious dose (EID_50_) of viruses harvested after inoculating 100 EID_50_ of each virus into 10-day-old SPF eggs and incubating at 37 °C for 72 h. To determine the EID_50_/mL, harvested viruses were serially diluted 10-fold (10^6^ to 10^9^-fold dilution), and each dilution was inoculated into five 10-day-old SPF eggs. After incubating the virus-inoculated eggs at 37 °C for 72 h, EID_50_/mL was calculated using the HA assay in each dilution and the Spearman-Karber method [36].

### Western blotting

To compare the band intensity of HA and NP proteins, western blot analysis was performed on recombinant viruses. The allantoic fluid from three recombinant viruses—22W_PR8, 22W_MVV, and 22W_KY—was loaded onto polyacrylamide gels (Bolt Bis-Tris Plus protein gels, 4–12%, Invitrogen) and transferred to nitrocellulose membranes using the iBlot 2 Gel Transfer Device (ThermoFisher). The membranes were blocked with 5% skim milk for 1 hour and then incubated with primary antibodies: chicken immune serum against 22W_MVV or Influenza A NP polyclonal antibody (Invitrogen, PA5-32242) for 1 hour. Secondary antibodies, Goat anti-Chicken IgG (IgY)-horseradish peroxidase (HRP) (BETHYL, TX, USA) or Goat anti-Rabbit IgG-HRP (BETHYL), were applied for 1 hour, followed by color development using 3,3’,5,5’-tetramethylbenzidine (TMB) substrate (SURMODICS IVD, MN, USA). The uncropped and unprocessed images of the western blot are provided in the Supplementary Fig. 4.

### Growth kinetics of recombinant virus in mammalian cell

To evaluate the potential infectious risk of the recombinant vaccine strains to mammals and humans, each virus was inoculated onto MDCK or Calu-3 cell lines at a 0.001 MOI. After one hour incubation at 37 °C in a 5% CO_2_, the inoculum was replaced with fresh media, and supernatants were harvested at intervals of 0, 24, 48, 72 and 96 h post inoculation. The harvested supernatants were subjected to 10-fold serial dilutions (10^1^ to 10^8^-fold dilution) and inoculated onto MDCK cells to determine the viral titer in TCID_50_/mL. The titer was calculated using HA assay results for each dilution and the Spearman-Karber method.

### Solid phase binding assay

To compare the receptor binding affinity of the recombinant viruses to α2,3-linked and α2,6-linked sialic acid, we conducted a solid phase binding assay based on a previous study [37]. Briefly, 96-well immunoplate (SPL, Pocheon-si, Republic of Korea) was coated with Fetuin (Sigma-Aldrich) at a concentration of 10ug/mL and incubated overnight at 4 °C. The next day, the Fetuin-coated immunoplate was washed three times with distilled water. The recombinant viruses, diluted to 16 HAU (in PBS with 2 µM neuraminidase inhibitor (Oseltamivir phosphate, Sigma-Aldrich)), were added to the washed plate and incubated overnight at 4 °C. The plate was then washed three times with PBS to remove virus, and it was blocked for 1 h using a blocking solution (PBS containing 0.1% neuraminidase (Sigma-Aldrich)-treated bovine serum albumin (BSA, MP Biomedicals)). After washing the plate three times with ice-cold PBS, then the serially diluted biotinylated sialylglycopolymers (Neu5Acα2-3Galβ1-4GlcNAcβ-sp3-PAA-biot, 3’SLN and Neu5Acα2-6Galβ1-4GlcNAcβ-sp3, 6’SLN) (GlycoNZ, New Zealand) were added and incubated for 1 h at 4 °C. The plate was washed three times, and horseradish peroxidase (HRP)-conjugated streptavidin (Invitrogen) was added, incubating for 1 h at 4 °C. The HRP was developed with the TMB substrate, and the reaction was stopped with 0.1M H_2_SO_4_ before measuring the absorbance at 450 nm using a microplate reader (TECAN, Switzerland).

### Chicken immune sera

To obtain immune sera against 21W_MVV and 22W_MVV, each virus was inactivated by treating it with a 37% formaldehyde solution (Sigma-Aldrich) at a 1:500 ratio to the virus solution. The mixtures were incubated at 37 °C for 24 h with continuous shaking. To confirm complete inactivation, formaldehyde-treated viruses were inoculated into 10-day-old SPF embryonated chicken eggs (ECEs), and the absence of the HA reaction with 1% cRBC was assessed 3 days post-inoculation. This process was repeated once more to ensure complete inactivation. The inactivated antigens were then emulsified with ISA78 (SEPPIC, France) in a 3:7 ratio to create an oil-emulsion vaccine. Each emulsion was administered intramuscularly at a dose of 0.5 mL to three-week-old SPF chickens (Namduck SPF, Republic of Korea), with five chickens per group. Sera were collected 3 weeks postvaccination.

### Virus inactivation

Formaldehyde inactivation was performed using the same method as previously described to inactivate the viruses. For binary ethylenimine (BEI) inactivation, 0.1 M BEI (Sigma-Aldrich) solution was added to the virus to achieve a final concentration of 1mM, and the mixture was incubated at 37 °C for 24 h with shaking. 1 M Sodium thiosulfate (DAEJUNG, Republic of Korea) was then added at 10 times the BEI concentration to neutralize it. For β-propiolactone (BPL) inactivation, a final concentration of 0.1% BPL (Tokyo Chemical Industry, Japan) was added, and the mixture was incubated at 4 °C for 16 h, followed by incubation at 37 °C for 1 h to ensure complete hydrolysis of BPL. Inactivation was verified by inoculating the inactivated virus preparation into five SPF eggs and checking for HA reactions after 3 days, a process that was repeated once more to ensure complete inactivation.

### Purification of virus or inactivated vaccine

Viruses or inactivated vaccine strains were concentrated by centrifugation at 50,000 g for 2 h in Type 70 Ti fixed-angle rotor (Optima XE-100, BECKMAN COULTER, CA, USA). Then, discontinuous 30-60% sucrose gradient was prepared, and the concentrated sample was layered on top, followed by centrifugation at 100,000 g for 2 h. A milky band, appearing at approximately the 40% sucrose level, was collected. The collected band was mixed with PBS to the maximum volume and centrifuged again at 50,000 g for 2 h to pellet the purified sample. The resulting pellet was resuspended in PBS, and the total protein amount was quantified using a bicinchoninic acid (BCA) protein assay kit (TAKARA, Japan). The samples were aliquoted and stored at -80 °C until further use.

### Cell internalization assay

To evaluate whether inactivated viruses retain their ability to mediate antigen entry into cells, in vitro cell internalization assays were conducted based on previous studies [38–40]. Live viruses were designated as positive controls, while media were used as negative controls. A549 cells were seeded in 24-well plates containing coverslips and cultured overnight at 37 °C in a 5% CO_2_. The next day, cell confluency was confirmed, and the cells were washed with PBS before 250 µL of live virus (64 HAU) or inactivated antigens (1024 HAU) was added to each well. After one hour incubation at 4 °C to synchronize virus binding, the cells were washed twice with PBS, replenished with fresh medium, and incubated at 37 °C for 4 h. Cells were then fixed with 4% paraformaldehyde (GeneAll Biotechnology, Songpa-gu, Republic of Korea), and washed twice with PBS. Permeabilization was performed using 0.1% Triton X-100 (Sigma-Aldrich) for 5 min at room temperature, followed by two PBS washes. After block the plate with blocking buffer (5% Normal goat serum (Vector laboratories, CA, USA) in PBST) the cells were incubated overnight at 4 °C with primary antibodies: Influenza A NP monoclonal antibody (Abcam, MA, USA, AB128193) or Influenza A M1 monoclonal antibody (Invitrogen, MA1-80736). The next day, cells were washed three times with PBS, and Alexa Fluor™ 488 (for NP protein) or 647 (for M1 protein)-labeled goat anti-mouse IgG (H + L) secondary antibodies (Invitrogen) were added for 1 h at room temperature in the dark room. DAPI solution (BioLegend, CA, USA) was used for nuclear staining, and coverslips were mounted on slides for observation under a confocal microscope (LSM 800, ZEISS, Oberkochen, Germany). Confocal images were visualized using ZEISS ZEN 3.9 software, with brightness and contrast adjustments uniformly applied across the entire image. Mean fluorescence intensity (MFI) measurements were performed as previously described using Fiji (ImageJ) [41]. Briefly, confocal images were processed by splitting the channels in ImageJ software to exclude the DAPI channel, followed by threshold adjustment to delineate cell boundaries. The edges of individual cells were then selected to measure MFI for each protein signal, with measurements taken from 10 cells per group. The final data were calculated by subtracting the mean MFI of the negative control group from the MFI values obtained for each group.

### Mouse experiments

To assess potential mammalian pathogenicity of vaccine strain in vivo, six-week-old female BALB/c mice (ORIENT BIO, Republic of Korea) were intranasally inoculated with 10^6^ EID_50_ of each virus under anesthesia with avertin (200 mg/kg) administered intraperitoneally (IP). Body weight changes were monitored in five mice per group for 13 days. Additionally, lungs from three mice per group were sampled on day 3 post-infection, homogenized using a TissueLyser II (QIAGEN, Hilden, Germany), and resuspended in PBS at a volume nine times the lung weight. After centrifugation, the supernatants were collected, and viral titers (TCID_50_/mL) were determined in MDCK cells.

To evaluate the immunogenicity and protective efficacy of whole inactivated virus (WIV) vaccines administered via intranasal routes, six-week-old female BALB/c mice were anesthetized via IP injection of avertin (200 mg/kg) and intranasally immunized with 5 µg/50 µL of vaccines inactivated with formaldehyde (F/A), binary ethylenimine (BEI), or beta-propiolactone (BPL) or PBS (Negative control). Two weeks after the initial vaccination, mice were boosted with the same vaccine using the same administration method. Three weeks post-boost, nasal wash (NW), bronchoalveolar lavage (BAL) fluid, and serum samples (*n* = 5) were collected for hemagglutination inhibition (HI), neuraminidase inhibition (NI), neutralization assays, and antigen-specific ELISA. To evaluate the protective efficacy of the vaccines, three weeks after the booster immunization, mice were anesthetized via IP injection of avertin (200 mg/kg) and challenged with 10 LD_50_ of either a genetically heterologous strain within the same subtype (A/Wild duck/Korea/SNU50-5/2009, H5N1) or a heterosubtypic strain (A/Puerto Rico/8/1934, PR8, H1N1). Five days post-challenge, lung and nasal turbinate tissues (*n* = 3) were collected to compare viral loads. Body weight changes and mortality rates were monitored for 14 days (*n* = 5), with a humane endpoint defined as 20% body weight loss. Mice reaching this endpoint were euthanized by carbon dioxide inhalation and included in mortality counts. Antigen-specific T-cell responses induced by the vaccines were evaluated five days after the challenge by isolating mononuclear cells from the spleen and lungs (*n* = 4–5) for flow cytometry analysis.

### Hemagglutination Inhibition (HI) assay

To evaluate antigenic changes associated with mutations, hemagglutination inhibition (HI) assay was performed based on the WHO-recommended standard protocols [42]. chicken immune sera were heat-inactivated at 56 °C for 30 min. Heat-inactivated sera were serially 2-fold diluted in a 96-well V-bottom plate and mixed with an equal volume of virus, each adjusted to 4 HAU. The mixtures were incubated at room temperature for 30 min, followed by the addition of 1% chicken red blood cells (cRBCs) and further incubation at room temperature at 4 °C for 40 min. The HI titer was recorded as the reciprocal of the highest serum dilution that completely inhibited hemagglutination of the cRBCs. To determine the HI titers induced by the intranasal administration of inactivated WIV vaccines, mouse serum was mixed with receptor-destroying enzyme (RDE, Denka Seiken, Japan) at a ratio of 1:3 and incubated at 37 °C for 18 h. The RDE was then inactivated by heating at 56 °C for 1 h. The RDE treated mouse serum was subsequently used for the HI assay.

### Neuraminidase Inhibition (NI) assay

For the NI titer measurement, the Enzyme-linked lectin assay (ELLA) was conducted using three different purified whole-virus antigens: the 22W_KY vaccine strain, SNU50-5, and PR8. The procedure for the ELLA followed previous descriptions [43]. Briefly, A 96-well immunoplate was coated with 25 µg/mL of Fetuin (Sigma-Aldrich) and incubated overnight at 4 °C. The following day, in a 96-well U-plate, heat-inactivated mouse serum was initially diluted 10-fold and then further subjected to 4-fold serial diultions using PBS + 2% BSA. The antigen diluted to its 90% effective concentration (EC_90_) was added to the 96-well U-plate and incubated at room temperature for 1.5 h. The Fetuin-coated plate was blocked with a blocking buffer (PBS + 2% BSA) at room temperature for 1 h and washed three times with PBST. After the reaction between antigen and serum was complete, the reaction mixture was transferred to the Fetuin-coated plate and incubated at 37 °C for 2 h. The plate was then washed three times with PBST, and 0.5 µg/mL Peanut agglutinin conjugated to HRP (PNA-HRP, Sigma Aldrich) was added to each well, followed by a 1.5-hour incubation in a dark room. Subsequently, the HRP was developed using the TMB substrate. The reaction was stopped using 0.1M H_2_SO_4_, and the absorbance at 450 nm was measured with a microplate reader (TECAN). The NI titer was represented as the serum dilution that achieved 50% inhibition of NA activity.

### Virus neutralization (VN) assay

For the VN titer measurement, RDE-treated mouse serum was two-fold diluted in infection medium consisting of DMEM supplemented with 1 µg/mL TPCK-treated trypsin, in a 96-well U-plate. The diluted serum was mixed with an equal volume of 100 TCID_50_ antigen and incubated at 37 °C for 1 h. Afterward, 100µL of a serum/antigen mixture was added to the MDCK cell and incubated for 72 h at 37 °C. At 72 h post-incubation, the supernatant was harvested and mixed in a 1:1 ratio with 1% cRBC. The VN titer was defined as the highest serum dilution that inhibited the hemagglutination of the cRBCs.

### Enzyme-linked immunosorbent assay (ELISA)

To evaluate IgG and IgA immune responses in serum, nasal wash (NW), and bronchoalveolar lavage (BAL) fluids induced by the intranasal administration of inactivated WIV vaccines, samples were analyzed using antigen-specific ELISA. Purified antigens were diluted to 1 µg/mL in PBS, and 50 µL of the antigen solution was coated onto each well of a 96-well immunoplate, which was then incubated overnight at 4 °C. The following day, the antigen-coated wells were blocked with blocking buffer (PBST + 1% BSA) to prevent non-specific binding and incubated at room temperature for 1 h. During the blocking step, serum, NW, and BAL samples were serially 4-fold diluted. After blocking, the plate was washed three times with PBST. Diluted serum, NW, and BAL samples were added at 50 µL per well and incubated at room temperature for 2 h. Following incubation, the plate was washed three times with PBST, and secondary antibodies (Goat anti-mouse IgG H&L-HRP, Abcam, or Goat anti-mouse IgA Cross-Adsorbed Secondary Antibody, Invitrogen) were added at 100 µL per well, followed by incubation at room temperature for 1 h. After 1 h incubation, HRP activity was developed using TMB substrate, and the reaction was stopped with 0.1 M H_2_SO_4_. Absorbance was measured at 450 nm using a microplate reader (TECAN). The area under the curve (AUC) was calculated using GraphPad Prism 9.5.1, with the dilution factor of the samples set as the x-axis values and the OD450 as the y-axis values. For the calculation, the baseline was set at 0.1, approximately twice the OD450 value of blank wells (containing no serum, NW, or BAL samples), and values below 0.1 were considered non-specific and excluded from the AUC calculation.

### Flow cytometry

To isolate cells from the lung and spleen, five days post challenge, mice were euthanized by carbon dioxide inhalation and perfused by cutting the abdominal artery and perfusing 10 mL of perfusion solution (PBS + 20 U/mL heparin solution, Sigma-Aldrich) through the right ventricle to clear blood from the lungs. The lungs were then dissected, ensuring all lobes were collected, and placed in complete RPMI on ice. Spleens were collected after trimming excess fat tissue and placed in complete RPMI on ice. For isolation splenocyte, spleens were pressed through 70 μm cell strainers using syringe plungers and centrifuged at 500 g for 5 min at room temperature. The resulting cell pellet was resuspended in 1 mL of RBC lysing buffer (Sigma-Aldrich) and incubated at room temperature for 2 min to lyse red blood cells. The reaction was terminated by adding 20 mL of FACS buffer, followed by centrifugation at 500 g for 5 min at room temperature. After removing the supernatant, the cell pellet was resuspended in 2.5 mL of complete RPMI, and cell counting was performed. Lung tissue was finely chopped and incubated in lung digestion solution containing 0.1 mg/mL DNase I (Sigma-Aldrich) and 1 mg/mL Collagenase D (Sigma-Aldrich) in RPMI-1640 at 37 °C with shaking for 1 h. The digested tissue was filtered through 100 μm strainers, with cold RPMI used to wash the cells through the filters. The cell suspension was centrifuged at 500 g for 5 min, and red blood cell lysis was performed. After lysis, the pellet was collected by centrifugation.

Lymphocytes from the lung were isolated using a Percoll gradient. The cell pellet was resuspended in 44% Percoll solution and gently layered onto 66% Percoll solution. The gradient was centrifuged and lymphocytes were collected from the interface between the 44% and 66% Percoll layers. The harvested lymphocytes were washed, counted, and resuspended in complete RPMI at appropriate concentrations for flow cytometry analysis. To stimulate splenocytes or lung cells, 96-well U-bottom plates were seeded with 2 × 10⁷ splenocytes/mL or 1 × 10⁷ lung cells/mL, dispensing 100 µL per well. Stimulation solution containing 40 µg/mL of purified PR8 or SNU50-5 antigen, 4 µg/mL Ultra-LEAF™ Purified anti-mouse CD28 Antibody (BioLegend, 102115), and 4 µg/mL Ultra-LEAF™ Purified anti-mouse CD49d Antibody (BioLegend, 103709) in complete RPMI was added at 50 µL per well. The plates were incubated at 37 °C in a 5% CO₂ incubator for 2 h. Brefeldin A (Invitrogen) was then added at 12 µg/mL (50 µL per well), followed by an additional 10–12 h of incubation. Stimulated cells were stained with LIVE/DEAD Fixable Near-IR dye (Invitrogen, L34975) in PBS at room temperature for 20 min in the dark and blocked with 1% Mouse BD Fc Block buffer (BD Biosciences, 553141) on ice for 15 min. Cells were then stained with CD3 (17A2, APC), CD4 (GK1.5, FITC), and CD8a (53 − 6.7, PE) monoclonal antibodies (eBioscience) in Fc Block buffer on ice for 30 min. For intracellular staining, cells were fixed and permeabilized (eBioscience) and stained intracellularly with Alexa Fluor^®^ 700 Rat Anti-Mouse IFN-γ (BD Bioscience, 557998) and PE/Dazzle™ 594 anti-mouse TNF-α (BioLegend, 506345) in permeabilization buffer (eBioscience) on ice for 30 min. Stained cells were collected using a SONY Cell Sorter (SH800S, SONY Biotechnology, USA) and analyzed with FlowJo v10 software. Fluorescence compensation was performed using UltraComp eBeads (Invitrogen). Fluorescence minus one (FMO) controls were used to gate IFN-γ- and TNF-α positive populations. The gating strategy and representative flow cytometry images are provided in Supplementary Fig. 6 and Supplementary Fig. 7, respectively.

### Statistic analysis

Statistical analyses were performed using GraphPad Prism 9.5.1. HI, NI, and VN titer values are presented as geometric mean titers (GMT) ± 95% confidence intervals (CI), while all other values are expressed as mean ± SD. Statistical significance between groups was determined using one-way or two-way ANOVA, followed by Tukey’s multiple comparison test or Dunnett’s multiple comparisons test as described in the figure legends. For the HI, NI, and VN assays specifically, titer values were converted to log_2_ for statistical analysis.

## Results

### Two distinct patterns observed in clade 2.3.4.4b H5N1 isolates from Asia during the early phase of emergence

**Fig. 1:**
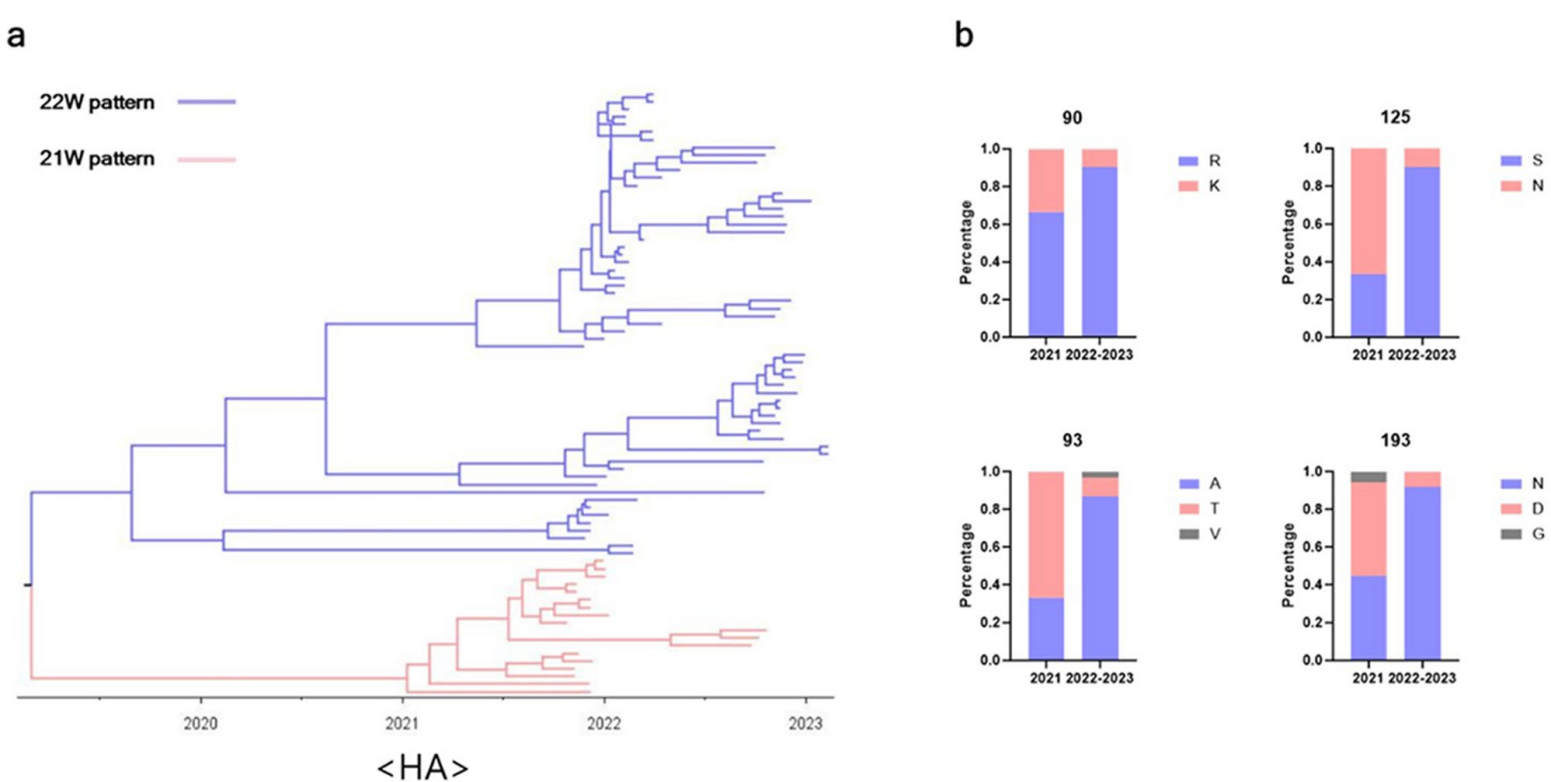
Time-scaled phylogenetic and genetic analysis of clade 2.3.4.4b H5N1 hemagglutinin (HA) amino acid sequences isolated from East Asia. (**a**) Time-scaled phylogenetic tree showing the evolutionary relationships of HA sequences of clade 2.3.4.4b H5N1 viruses (*n* = 79) in East Asia collected between October 2021 and February 2023. The tree highlights two distinct branches, with one pattern presented in blue and the other in pink. (**b**) Comparison of the prevalence of specific amino acid residues at four distinct positions (90, 93, 125, and 193) between the viruses with the blue pattern and those with the pink pattern

We obtained HA sequences from 79 clade 2.3.4.4b H5N1 HPAIV isolates collected in East Asia between October 2021 and February 2023 from the Global Initiative for Sharing All Influenza Data (GISAID) database. The temporal evolutionary trends of these HA sequences were analyzed using the Bayesian Evolutionary Analysis Sampling Trees (BEAST) v1.10.4 software. Analysis of the time-scaled phylogenetic tree revealed that during the initial phase of clade 2.3.4.4b H5N1 emergence in 2021, the virus diverged into two distinct patterns: a blue pattern (22 W pattern) and a red pattern (21 W pattern), with the blue pattern becoming dominant after 2022 (Fig. 1a). Comparative analysis of HA sequences revealed key differences at four positions that distinguish the two distinct patterns: residues 90, 93, 125, and 193 (H3 numbering). As shown in Fig. 1b, in 2021, mixed patterns of arginine or lysine (R or K) at residue 90, alanine or threonine (A or T) at residue 93, serine or asparagine (S or N) at residue 125, and asparagine or aspartic acid (N or D) at residue 193, respectively, were oberved, but transitioned from 2022 to 2023 to a dominant pattern of R90/A93/S125/N193. To broaden the analysis beyond East Asia, additional clade 2.3.4.4b H5N1 sequences isolated from Europe (*n* = 165) and North America (*n* = 83) between October 2021 and February 2023 were included (Supplementary Fig. 1). Analysis of the time-scaled phylogenetic tree revealed that isolates from Europe and North America consistently presented the R90/A93/S125/N193 (22 W pattern) sequence after the emergence of clade 2.3.4.4b H5N1. Notably, only viruses from East Asia presented the K90/T93/N125/D193 sequence pattern (21 W pattern).

### Heat stability enhancement of the 22W_MVV induced by the R90K mutation in hemagglutinin

**Table 1:** Genome constellation and 50% egg infectious dose titers of Recombinant H5N1 viruses in embryonated chicken eggs (ECEs)

Recombinant virus	HA	NA	PB2	PB1	PA	NP	M	NS	EID_50_/mL^a^
22W_PR8	22 W HA^b^	22 W NA	PR8	PR8	PR8	PR8	PR8	PR8	8.91 ± 0.14
22W_MVV	22 W HA	22 W NA	310-MVV^c^	PR8	PR8	PR8	PR8	PR8	9.91 ± 0.14 ***
22W_KY	22 W HA-R90K H110Y^b^	22 W NA	310-MVV	PR8	PR8	PR8	PR8	PR8	9.91 ± 0.14 ***

^a^50% egg infectious dose (EID_50_/ml) of recombinant viruses harvested after inoculating 100 EID_50_ of each recombinant virus into 10-day-old SPF embryonated chicken eggs. The data are presented as the mean ± standard deviation (SD) of three independent experiments

^b^The HA cleavage site was attenuated from RERRRKR to ASGR

^c^PB2 gene of A/chicken/Korea/01310/2001 (01310, H9N2) virus with I66M, I109V and I133V mutations

^*^Significantly different from the virus titer of 22W_PR8 (***p < 0.001), Statistical significance was calculated by one-way ANOVA with Tukey’s multiple comparisons test

On the basis of the phylogenetic analysis, consensus HA and NA sequences (22 W HA and 22 W NA, respectively) were generated from 3,004 clade 2.3.4.4b H5N1 virus isolates collected in East Asia, Europe, and North America between October 2021 and February 2023. Additionally, an HA sequence with R90K/A93T/S125N/N193D mutations introduced into the 22 W HA sequence (21 W HA) was generated. To generate recombinant viruses, these sequences were combined with six internal genomic backbone segments (PB2, PB1, PA, NP, M, and NS) from PR8, as well as a PB2 gene from 01310 containing the I66M, I109V, and I133V (MVV) mutations, which we previously found to increase replication efficiency and reduce mammalian infectivity in Y280-lineage H9N2 vaccine strains (Table 1) [19]. To compare the effects of individual HA mutations, recombinant viruses containing single mutations (R90K, A93T, S125N, or N193D) were also generated (Supplementary Table 1).

**Fig. 2:**
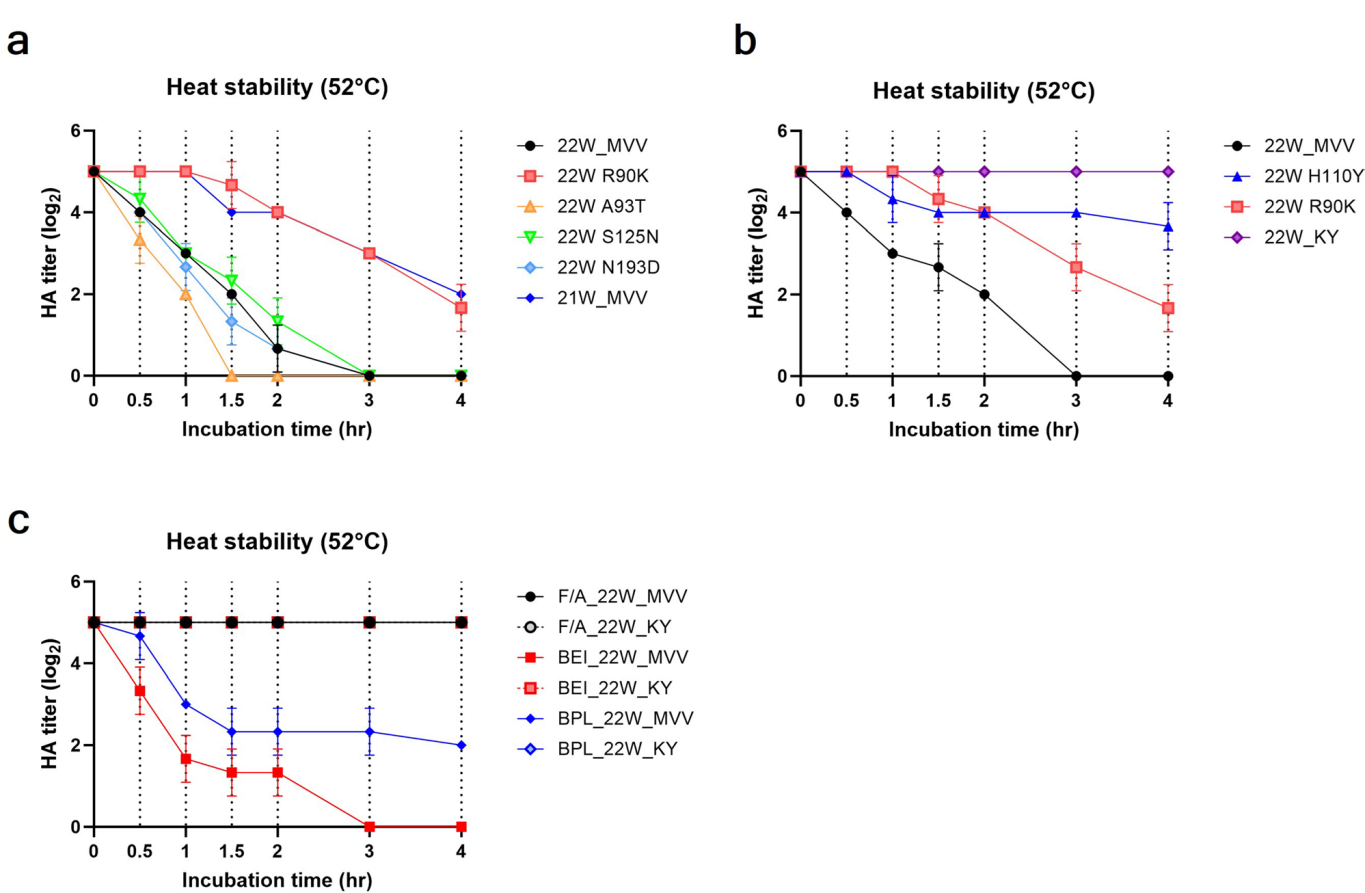
Heat stability of the recombinant and inactivated H5N1 strains. Each recombinant virus or inactivated virus was diluted to 32 HAU and incubated at 52 °C for 0.5, 1, 1.5, 2, 3, or 4 h. After heat treatment, the HA titer of each virus was measured and recorded. The HA titers were plotted over time after heat treatment for three sets of comparisons: (**a**) among 22W_MVV, 21W_MVV, and recombinant viruses with individual HA mutations (R90K, A93T, S125N, and N193D) and (**b**) among 22W_MVV, 22W_R90K, 22W_H110Y, and 22W_KY and (**c**) among formaldehyde (F/A)-inactivated 22W_MVV and 22W_KY, binary ethylenimine (BEI)-inactivated 22W_MVV and 22W_KY, and β-propiolactone (BPL)-inactivated 22W_MVV and 22W_KY. Data for each HA titer data are presented as the mean ± standard deviation (SD) of two independent triplicate experiments

Compared with the 21W_MVV virus, the 22W_MVV virus exhibited considerably lower heat stability (Fig. 2a). Among the individual mutations tested, only the HA-R90K mutation significantly enhanced heat stability, which was to a level comparable to that of the 21W_MVV strain. We also evaluated whether the HA-H110Y mutation, which we previously showed to increase heat stability in H5 subtype influenza viruses, could have a similar effect on the 22W_MVV virus [44]. Indeed, HA-H110Y alone improved heat stability, and importantly, combining the R90K and H110Y mutations (22W_KY) resulted in a synergistic increase in thermal stability and maintained HA activity without measurable reduction even after 4 h at 52 °C (Fig. 2b). These findings indicate that the 22W_KY virus shows promise as a vaccine strain with superior stability and preservation characteristics. Additionally, we further examined the thermal stability of both 22W_MVV and 22W_KY after inactivation by three different substances—formaldehyde (F/A), binary ethylenimine (BEI), and β-propiolactone (BPL) (Fig. 2c). Consistent with the live virus results, all three inactivation methods maintained the high thermal stability of the 22W_KY virus at 52 °C. Notably, F/A-inactivated 22W_MVV also showed no reduction in HA titers over the 4 h. When 22W_MVV was inactivated with BPL, the HA titer declined gradually over time but remained at a measurable level after 4 h. In contrast, the HA activity of 22W_MVV inactivated with BEI was completely lost after 3 h at 52 °C, the same as that of the live 22W_MVV virus.

### Reduction in receptor binding affinity and altered antigenicity of the 22W_MVV virus induced by the N193D mutation

A solid-phase receptor binding assay revealed that the 22W_MVV virus exhibited significantly higher binding affinity for α2,3-linked sialic acid (2,3-SA) than did the 21W_MVV virus (Supplementary Fig. 2a). No significant changes in the receptor binding affinity of the recombinant viruses with R90K, A93T, and S125N mutations were observed. However, the N193D mutation led to a reduction of the affinity of the 22W_MVV virus for 2,3-SA. Negligible binding to the α2,6-linked receptor was observed for all recombinant H5N1 viruses (Supplementary Fig. 2b).

**Table 2:** Cross hemagglutination Inhibition (HI) assay results comparing antigenicity of Recombinant H5N1 viruses with mutations at residues 90, 93, 110, 125, and 193

Immune serum	Geometric mean HI antibody titer						
	Antigen						
	21W_MVV	22W_MVV	22 W R90K	22W_KY	22 W A93T	22 W S125N	22 W N193D
21W_sera	1351.2	194.0^*^	111.4^**^	194.0^*^	168.9^*^	194.0^*^	1351.2
22W_sera	1351.2	1176.3	891.4	1351.2	891.4	1351.2	1176.3
Negative	< 1	< 1	< 1	< 1	< 1	< 1	< 1

^*^ Statistical significance of HI titers compared to those against 21W_MVV antigen (*p < 0.05, **p < 0.01). HI titer values were converted to log_2_, and statistical analysis was performed using one-way ANOVA with Tukey’s multiple comparisons test

To evaluate the antigenic differences among viruses with R90K, A93T, H110Y, S125N, and N193D mutations, a cross-hemagglutination inhibition (HI) assay was performed (Table 2). Consistently high HI responses were observed in the cross-HI titer comparison for immune sera obtained from the 22W_MVV immunization (22W_sera), with no significant differences detected across all tested antigens variants (21W_MVV, 22W_MVV, 22 W R90K, 22W_KY, 22 W A93T, 22 W S125N, and 22 W N193D). However, immune sera obtained from 21W_MVV immunization (21W_sera) presented significantly lower cross-HI titers against the 22W_MVV, 22 W R90K, 22W_KY, 22 W A93T, and 22 W S125N antigens than against the homologous 21W_MVV antigen. Notably, the HI titer against the 22 W N193D antigen was comparable to that against the 21W_MVV antigen. Overall, mutations at residues 90, 93, 110 and 125 had minimal impacts on the antigenicity of the virus. However, sera generated against the D193-containing strain (21W_sera) had significantly reduced immunogenicity against N193-containing antigens (including 22W_MVV, 22 W R90K, 22W_KY, 22 W A93T, and 22 W S125N). These findings suggest that D193-containing strains are susceptible to immunogenic alterations caused by a single mutation at residue 193, underscoring their vulnerability as vaccine candidates.

### Increased replication and antigen production in embryonated chicken eggs (ECEs) induced by incorporating 310-MVV PB2 into recombinant H5N1 vaccine strains

To evaluate whether 310-MVV PB2 gene, which previously increased the replication efficiency of recombinant Y280-lineage H9N2 vaccine strains [19], also contributes to increased replication when combined with the HA and NA genes of clade 2.3.4.4b H5N1, we compared the replication efficiency and HA antigen yield of 22W_PR8 (containing PR8-PB2), 22W_MVV, and 22W_KY in ECEs.

**Fig. 3:**
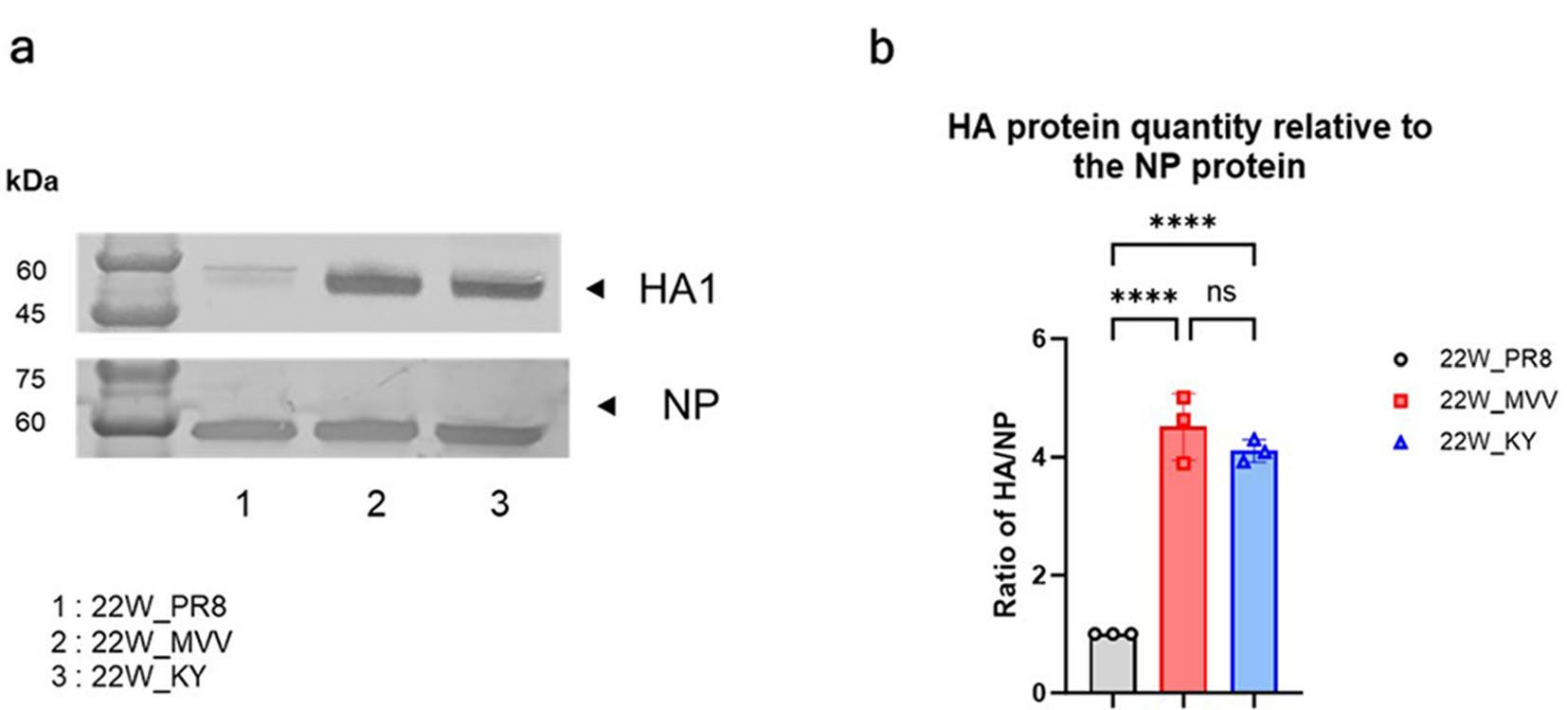
Comparison of antigen levels of recombinant H5N1 viruses by western blot analysis. Allantoic fluid from each recombinant H5N1 vaccine strain was loaded onto polyacrylamide gels. (**a**) Western blotting analysis was then performed using 22W_sera, which was also used in the cross-HI assay, for HA and the anti-NP pAb for the NP to visualize the levels of HA or NP antigens present in each virus. (**b**) To quantify the HA1 antigen, the intensity of the HA1 band relative to the NP band intensity was evaluated using ImageJ software. The HA1 and NP band intensities of 22W_PR8 were set as a baseline with a value of 1, and the relative HA1 band intensities, normalized to the NP band intensities, are presented as the mean ± SD from triplicate experiments. Statistical significance was analyzed using one-way ANOVA, followed by Tukey’s multiple comparison test, with significant differences indicated by asterisks (****p < 0.0001, ns: not significant)

The replication efficacies of vaccine strains containing 310-MVV PB2 gene (22W_MVV and 22W_KY) in ECEs were 10^9.91 ± 0.14^ EID₅₀/mL, which were 10-fold higher and significantly greater than that of 22W_PR8 containing PR8 PB2 gene (10^8.91 ± 0.14^ EID₅₀/mL) (Table 1). In addition to evaluating replication efficiency in ECEs, western blot analysis was performed on recombinant H5N1 vaccine strains to compare the antigen yield of the HA protein (Fig. 3a). The NP band intensities were comparable across 22W_PR8, 22W_MVV, and 22W_KY; however, the HA1 band intensity of 22W_PR8 was considerably lower than those of 22W_MVV and 22W_KY (Supplementary Fig. 3). Analysis of the relative band intensity revealed that the HA/NP ratios of 22W_PR8 were 4.51-fold and 4.1-fold lower than those of 22W_MVV and 22W_KY, respectively (Fig. 3b). No significant difference in the HA/NP ratio was observed between 22W_MVV and 22W_KY. Overall, these results indicate that the 310-MVV PB2 gene contributes primarily to increased replication efficiency and antigen production yield in our recombinant H5N1 vaccine strains, whereas the HA-R90K H110Y mutation does not significantly impact antigen levels.

### The 310-MVV PB2 and R90K/H110Y mutations reduce the risk of mammalian infection

**Fig. 4:**
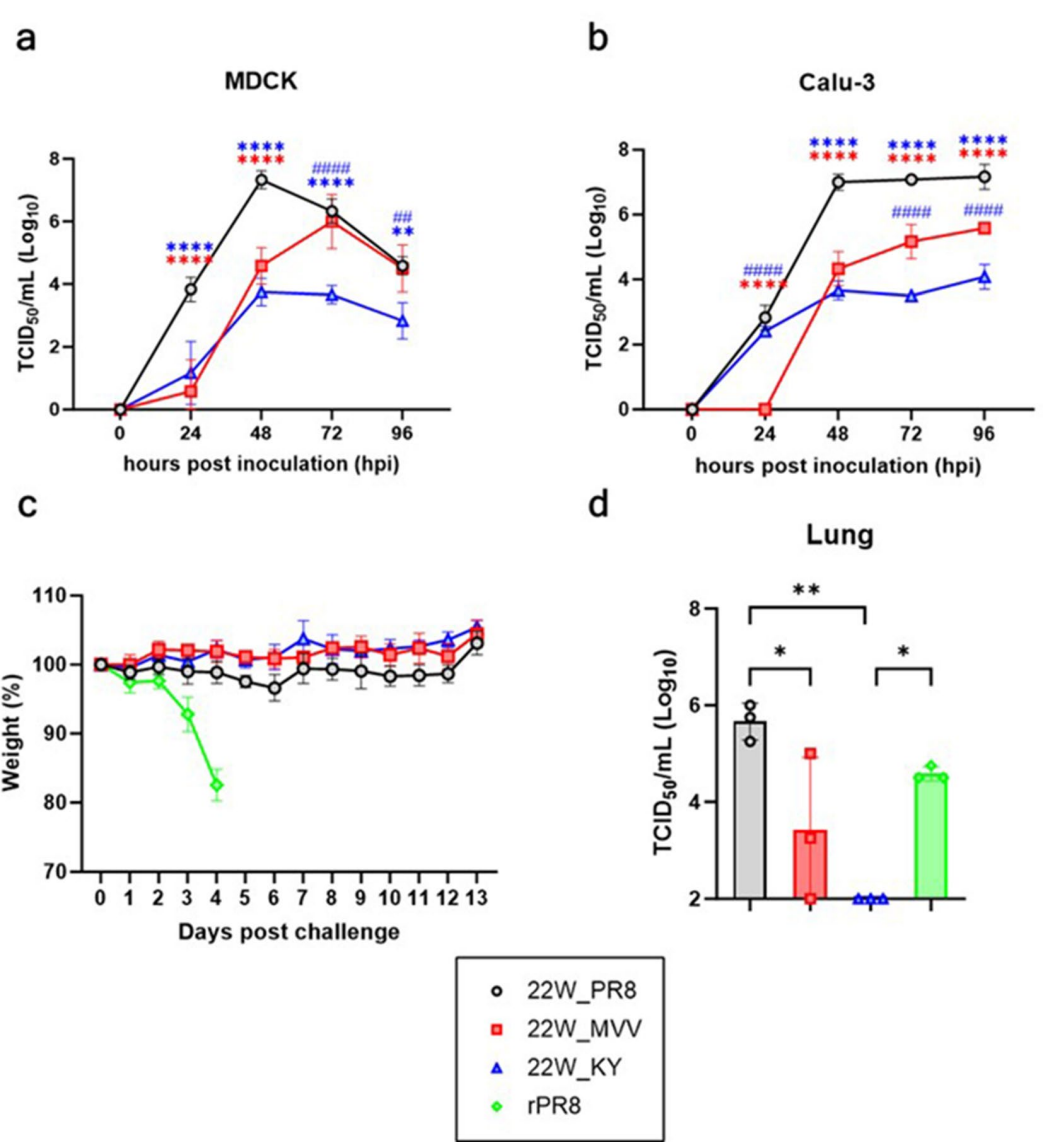
Evaluation of the safety profile of recombinant H5N1 vaccine strains in in vitro and in vivo models. The growth kinetics of recombinant H5N1 viruses (22W_PR8, 22W_MVV, and 22W_KY) were evaluated in mammalian (MDCK) and human respiratory (Calu-3) cells. Recombinant H5N1 viruses were inoculated at a multiplicity of infection (MOI) of 0.001 TCID_50_/cell into (**a**) MDCK cells and (**b**) Calu-3 cells. Viral supernatants were harvested at 0, 24, 48, 72, and 96 h postinoculation, and virus titers at each time point were determined and are presented as the mean ± SD of TCID_50_/mL values from triplicate experiments for each group. Statistical significance was determined using two-way ANOVA, followed by Tukey’s multiple comparison test. The blue asterisks indicate statistical significance between 22W_PR8 and 22W_KY, whereas the red asterisks represent significance between 22W_PR8 and 22W_MVV. The blue hash symbols (#) denote significant differences between 22W_MVV and 22W_KY (**p < 0.01, ##*p* < 0.01, ****p < 0.0001, ####*p* < 0.0001). (**c**) Body weight changes in recombinant H5N1 virus-infected mice were monitored. Groups of five six-week-old female BALB/c mice were intranasally inoculated with 10⁶ EID_50_/50 µL of each virus, and body weight changes were recorded for 13 days. (**d**) Lung virus titers (TCID_50_/mL) were measured at 3 days postinfection (dpi) in mice infected with each virus (*n* = 3 per group). The lung virus titer data are presented as mean ± SD, and statistical significance was determined using one-way ANOVA, followed by Tukey’s multiple comparison test. Significant differences are indicated by asterisks (*p < 0.05, **p < 0.01)

To assess the risk of mammalian and human infection by our recombinant H5N1 vaccine strains, replication efficiency was compared in mammalian (Madin-Darby canine kidney (MDCK)) and human respiratory (Calu-3) cell lines. In MDCK cells, the highest replication efficiency was observed for 22W_PR8, with significantly faster growth at 24 h; growth peaked at 48 h and subsequently declined (Fig. 4a). In contrast, the titers of 22W_MVV were significantly lower than those of 22W_PR8 during the first 48 h, but 22W_MVV titers peaked at 72 h, after which they remained comparable to those of 22W_PR8. Significantly lower replication efficiency of 22W_KY was observed throughout the time points, with reduced titers relative to 22W_MVV after 72 h. In Calu-3 cells, superior replication efficiency of 22W_PR8 was also observed, with efficient growth that peaked at 48 h and high titers maintained afterward (Fig. 4b). Significantly lower titers of both 22W_MVV and 22W_KY than of 22W_PR8 were observed at all time points. While higher titers of 22W_KY than of 22W_MVV were initially observed at 24 h, 22W_KY and 22W_MVV titers were comparable at 48 h; after 72 h, 22W_KY titers were significantly lower than 22W_MVV titers.

To further evaluate the potential pathogenicity and infectivity of recombinant H5N1 vaccine strains in mammalian species, BALB/c mice were challenged with each vaccine strain. No weight loss or mortality was observed in any group except for the rPR8 virus control group, indicating no detectable pathogenicity in mice for any of our recombinant H5N1 vaccine strains (Fig. 4c). However, lung titers of 22W_PR8 were significantly higher than those of 22W_MVV and 22W_KY and even surpassed the lung titers observed for the rPR8 virus (Fig. 4d). The 22W_MVV virus was detectable in the lungs of two out of three mice, whereas the 22W_KY virus was completely undetectable in the lungs. Overall, these findings indicate that while the 22W_PR8 virus had the highest potential for infectivity in mammalian hosts, the 22W_KY virus had the lowest viral fitness in mammalian systems. These findings suggest that the 310-MVV PB2 and HA-R90K/H110Y mutations collectively contribute to reduced replication efficiency in mammalian cells, making the 22W_KY virus a promising vaccine candidate with the lowest risk of mammalian infection.

In conclusion, 22W_KY vaccine strain had significantly higher stability than its parental 22W_MVV vaccine strain while maintaining high production efficiency, with no detectable changes in antigenicity. Furthermore, the risk of mammalian infection was confirmed to be lower with the 22W_KY vaccine strain than with the 22W_PR8 vaccine strain. These attributes support the selection of the 22W_KY vaccine strain as the final clade 2.3.4.4b H5N1 WIV vaccine strain for efficacy evaluation as an intranasal vaccine.

### The cell internalization of the 22W_KY vaccine strain inactivated with BEI was more efficient than that of strains treated with F/A or BPL

**Fig. 5:**
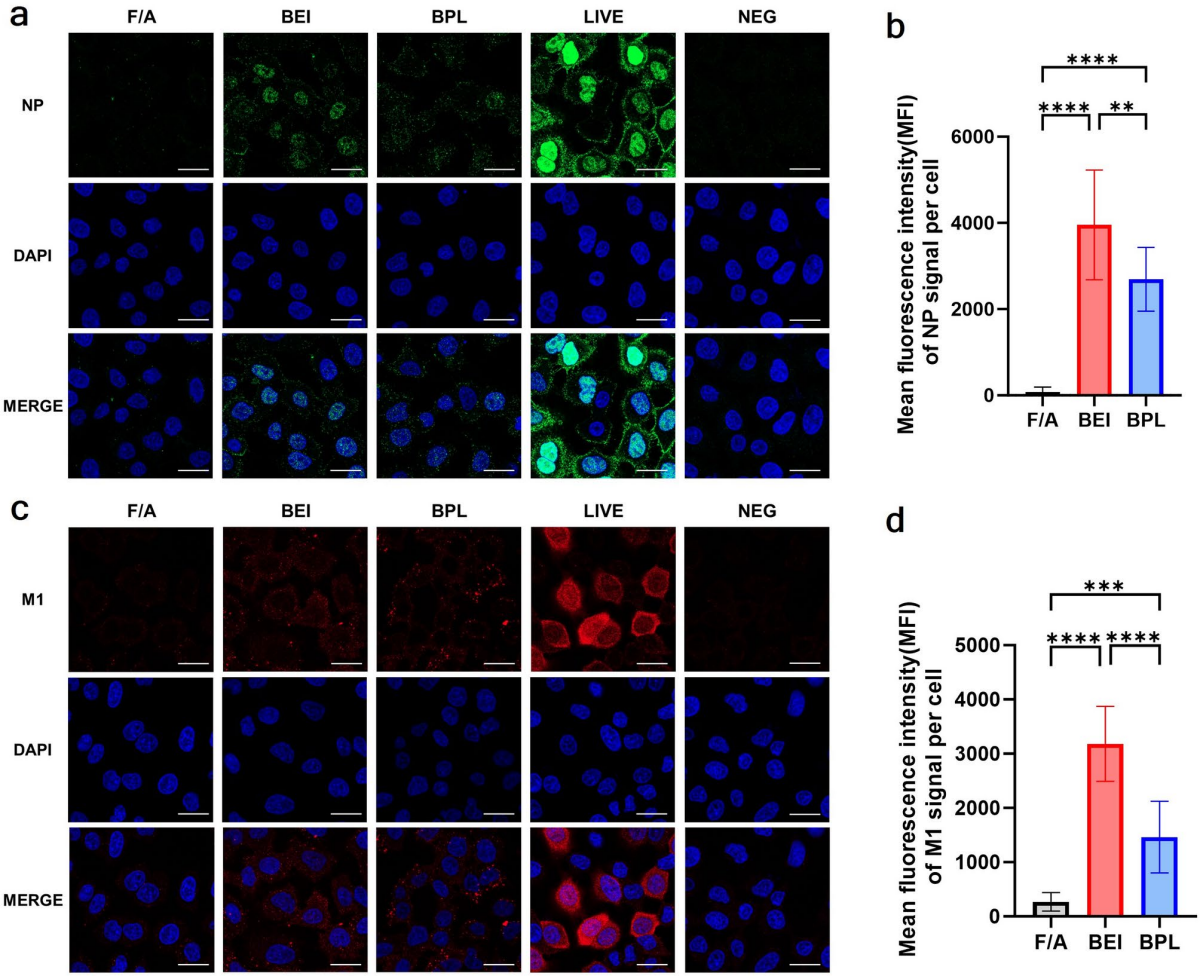
Analysis of the cellular entry and distribution of NP and M1 proteins of inactivated and live 22W_KY. Confocal microscopy images showing the distribution of (**a**) NP protein and (**c**) M1 protein in A549 cells following treatment at 37 °C for 4 h with formaldehyde-inactivated (F/A), BEI-inactivated, BPL-inactivated, live 22W_KY (positive control), or media-only (negative control). NP protein was labeled with Alexa Fluor 488 (green), the M1 protein was labeled with Alexa Fluor 647 (red), and the nuclei were stained with DAPI (blue). Scale bars represent 20 μm. The quantification of the mean fluorescence intensity (MFI) for (**b**) NP and (**d**) M1 signals per cell was performed by selecting the edges of individual cells to measure the MFI for each protein signal. Measurements were obtained from 10 cells per group, and final values were calculated by subtracting the mean MFI of the negative control group from the MFI values of each treatment group. The data are presented as the mean ± SD, and statistical significance was determined using one-way ANOVA, followed by Tukey’s multiple comparison test (***p < 0.001, ****p < 0.0001, ns: not significant)

To be effective as a mucosal vaccine, an antigen must efficiently penetrate respiratory mucosal epithelial cells to stimulate the immune system, such as mucosa-associated lymphoid tissues (MALTs), thus eliciting a robust immune response [24, 45]. To evaluate whether influenza virus retains its ability to enter cells after inactivation, as well as to determine the most effective inactivation method, we compared the extent of cellular entry of the WIV into respiratory cells using three different inactivation methods. A549 cells were inoculated with either live 22W_KY or F/A-inactivated 22W_KY (F/A_22W_KY), BEI-inactivated 22W_KY (BEI_22W_KY), or BPL-inactivated 22W_KY (BPL_22W_KY) and incubated at 37 °C for 4 h to assess cell entry. The levels of NP and M1 proteins within cells were tracked to compare the extent of cell entry. Confocal imaging findings confirmed that a substantial amount of live 22W_KY NP protein was present in both the cytoplasm and nucleus after 4 h, with significant amount of M1 protein observed in the cytoplasm as well (Fig. 5a, c). Varying levels of cell entry of WIVs were observed depending on the inactivation method used (Fig. 5). Confocal imaging and mean fluorescence intensity (MFI) analysis revealed that a minimal amount of F/A_22W_KY entered cells. In contrast, the NP and M1 signals indicated a greater amount of BPL_22W_KY within cells than F/A_22W_KY. Notably, among all WIV groups, the highest level of intracellular entry was observed for BEI_22W_KY, confirming its superior effectiveness in penetrating cells. These findings indicate that sufficient receptor-binding activity and membrane fusion capability of the HA protein of BEI_22W_KY is retained to facilitate entry into the cell. Furthermore, the inactivation method used significantly influences the cellular entry and distribution of inactivated 22W_KY protein, with the highest efficiency observed for BEI inactivation among the methods tested.

### The protective efficacy against lethal challenges with heterologous and heterosubtypic viruses of the BEI-inactivated vaccine strain administered intranasally was superior to that of vaccine strains treated with F/A or BPL

**Fig. 6:**
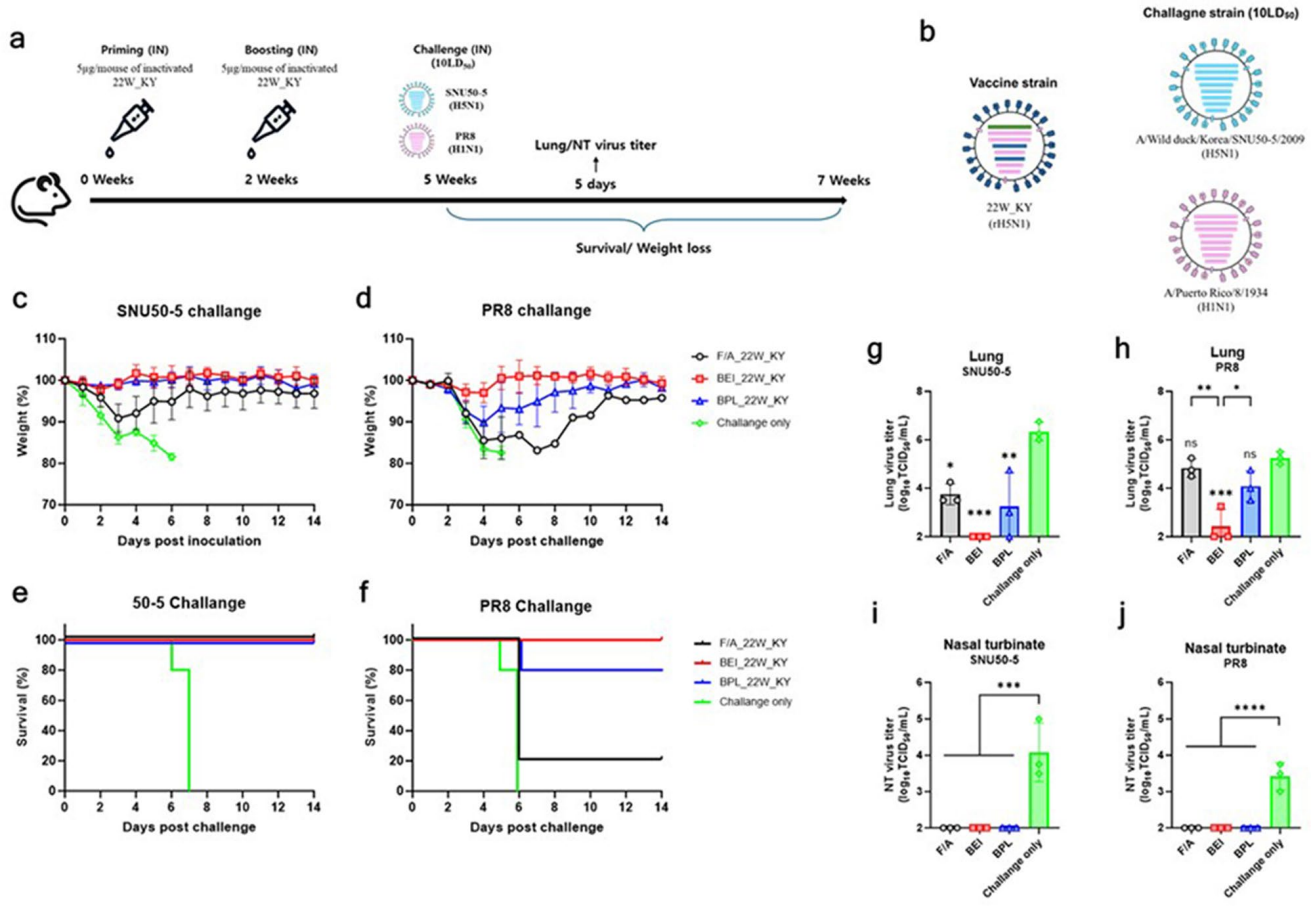
Intranasally administered 22W_KY inactivated with binary ethylenimine provides robust protection efficacy against heterologous and heterosubtypic virus challenges in mice. (**a**) Schematic representation of the experimental procedure for immunization and challenge studies. Groups of eight six-week-old BALB/c mice were intranasally immunized with 5 µg/50 µL 22W_KY inactivated with formaldehyde (F/A), binary ethylenimine (BEI), beta-propiolactone (BPL) or PBS (negative control). Two weeks after priming immunization, the mice received a booster immunization via the same route. Three weeks after the booster immunization, the mice were challenged with 10 LD_50_ of either heterologous A/Wild duck/Korea/SNU50-5/2009 (SNU50-5, H5N1) or heterosubtypic A/Puerto Rico/8/1934 (PR8, H1N1) virus. (**b**) Schematic representation of the genome composition of the 22W_KY vaccine strain, the SNU50-5 virus, and the PR8 virus. Each bar represents a genomic segment in descending order of length, corresponding to PB2, PB1, PA, HA, NP, NA, M, and NS. The color coding indicates the origin of each segment: green: 310-MVV PB2 segment; blue: HA and NA segments of 22W_KY; pink: PR8 virus segments; light blue: SNU50-5 virus segments. The SNU50-5 virus shares the same subtype as the vaccine strain but exhibits antigenic differences, whereas the PR8 virus, despite being a different subtype, shares identical internal genes with the vaccine strain, except for the PB2 segment. (**c-f**) Body weight changes and survival rates were monitored for 14 days postchallange (*n* = 5). (**g-j**) Viral loads in lung and nasal turbinate (NT) tissues were assessed five days postchallange to determine the effectiveness of each vaccine in reducing virus replication (*n* = 3). The data are presented as the mean ± SD, and statistical significance was determined by one-way ANOVA, followed by Tukey’s multiple comparison test. Asterisks directly above the bars indicate statistical significance compared with the negative control (*p < 0.05, **p < 0.01 ***p < 0.001, ****p < 0.0001)

In accordance with the experimental design shown in Fig. 6a, BALB/c mice were intranasally inoculated with 22W_KY inactivated with F/A, BEI, or BPL. Three weeks after the booster vaccination, the mice were challenged with lethal doses of either the heterologous A/Wild duck/Korea/SNU50-5/2009 (SNU50-5, H5N1) virus or the heterosubtypic A/Puerto Rico/8/1934 (PR8, H1N1) virus to assess the protective efficacy of each inactivated 22W_KY intranasal vaccine. The SNU50-5 virus is a low pathogenic avian influenza H5N1 strain that is the same subtype as the vaccine strain but has antigenic differences. In contrast, the PR8 virus belongs to a different subtype but has internal genes identical to those of the vaccine strain, except for the PB2 segment (Fig. 6b). The HA1 of the SNU50-5 virus shares 84.6% amino acid identity with that of 22W_KY, whereas its NA shows 97.2% homology; the HA1 of the PR8 virus shares 55.4% identity with that of 22W_KY, while the NA shows 83.5% homology (Supplementary Table 2).

Following challenge with the SNU50-5 virus, severe weight loss and 100% mortality within 7 days were observed in control group (challenge only) mice (Fig. 6c, e). In contrast, stable body weight and 100% survival of mice immunized with BEI_22W_KY or BPL_22W_KY were observed over the 14-day observation period, indicating strong protective efficacy. For mice immunized with F/A_22W_KY, 100% survival was also observed, although transient weight loss was observed up to 3-day post-challenge, followed by a gradual recovery of body weight. This findings indicates that BEI_22W_KY and BPL_22W_KY provided protection against the SNU50-5 virus that was superior to that of F/A_22W_KY. Following challenge with the heterosubtypic PR8 virus, rapid weight loss and 100% mortality were observed in control group (challenge only) by day 6 (Fig. 6d, f). The weight loss of mice immunized with F/A_22W_KY was similar to that of control group mice, and 80% mortality rate was observed in mice immunized with F/A_22W_KY, indicating limited protective efficacy. Up to 10% weight loss was observed on day 3 in mice vaccinated with BPL_22W_KY, but body weight subsequently recovered, resulting in 80% survival. No measurable weight loss and 100% survival were observed in mice vaccinated with BEI_22W_KY indicating strong protective efficacy of BEI_22W_KY against the PR8 virus. These findings suggest that BEI_22W_KY and BPL_22W_KY provide protection as inactivated mucosal vaccines that is superior to that provided by F/A_22W_KY, with the most robust protective effect against heterosubtypic viral challenges being demonstrated for BEI_22W_KY.

The viral loads in the lungs and nasal turbinates (NTs) also varied depending on the inactivation method employed. Following SNU50-5 challenge, high viral loads were observed in the lungs and NTs of the control group (Fig. 6g, i). Compared with the control group, significantly reduced viral loads were observed in all the vaccinated groups, with complete virus clearance observed in the NTs of all groups. However, differences in virus clearance were noted in the lungs. All three mice immunized with F/A_22W_KY had measurable viral loads, whereas two out of three mice immunized with BPL_22W_KY still had measurable viral loads. In contrast, complete virus clearance was achieved in all mice immunized with BEI_22W_KY indicating that BEI_22W_KY provided the highest level of protection. After the PR8 challenge, high viral loads were also observed in the lungs and NTs of the control group (Fig. 6h, j). Complete virus clearance was observed in the NTs of all vaccinated groups. The viral loads of mice vaccinated with F/A_22W_KY and BPL_22W_KY were not significantly different from those of control group mice, suggesting limited viral clearance capacity of F/A_22W_KY and BPL_22W_KY. In contrast, mice vaccinated with BEI_22W_KY had significantly lower viral loads than mice vaccinated with the F/A_22W_KY and BPL_22W_KY, with no measurable virus loads in two out of three mice, indicating that BEI_22W_KY is the most effective vaccine for viral clearance.

### Lack of HI response and comparable NI and VN responses across vaccine groups

**Fig. 7:**
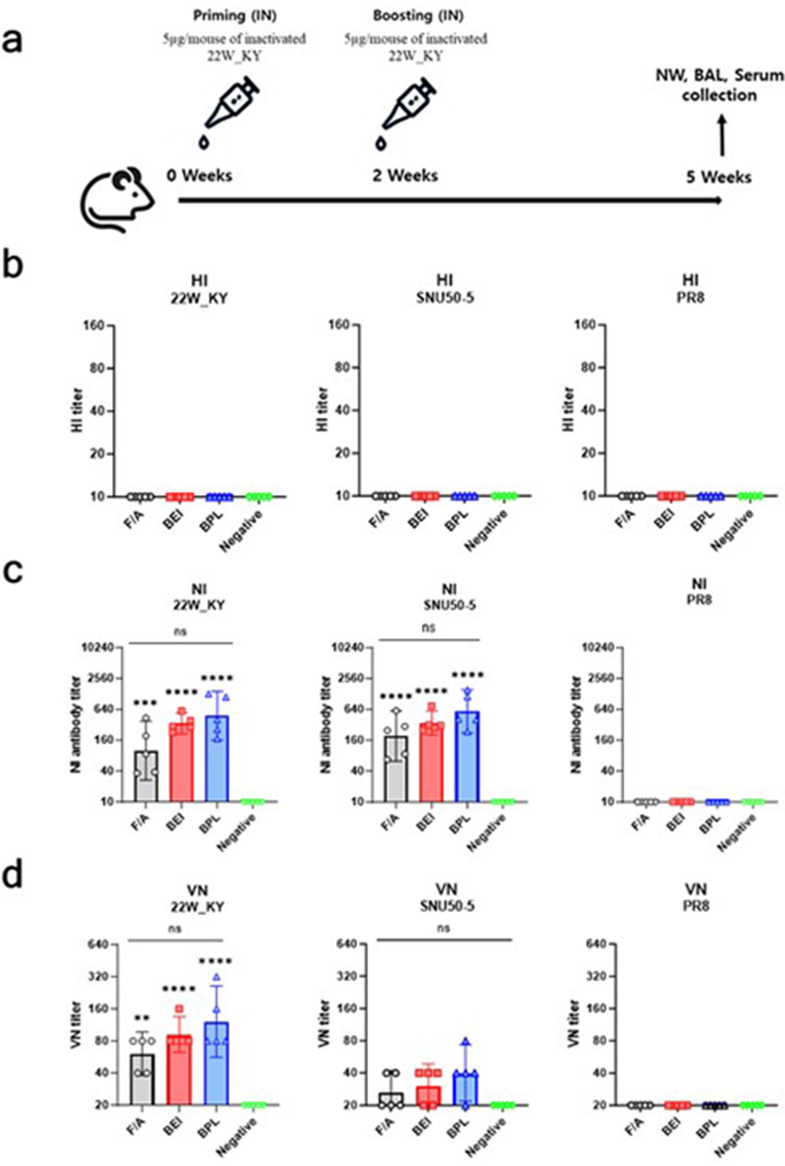
Evaluation of serum responses (HI, NI, and VN) induced by intranasal administration of the inactivated 22W_KY virus in mice. (**a**) Schematic representation of the experimental design. Groups of five six-week-old BALB/c mice were intranasally immunized with 22W_KY inactivated with F/A, BEI, or BPL, followed by booster immunization. Three weeks after the booster immunization, nasal wash (NW), bronchoalveolar lavage (BAL), and serum samples were collected for immunological assays. (**b-d**) Hemagglutination inhibition (HI), neuraminidase inhibition (NI), and virus neutralization (VN) assays were performed using collected serum samples to assess the immune responses against the vaccine strain 22W_KY, as well as the SNU50-5 and PR8 antigens. All the data are presented as the geometric mean titer (GMT) ± 95% confidence interval (CI). Statistical significance was analyzed using one-way ANOVA with Tukey’s multiple comparisons test after all titer values were converted to log_2_ values. Significant differences are denoted by asterisks, with asterisks displayed directly above the bars indicating statistical significance compared with the negative control (**p < 0.01, ***p < 0.001, ****p < 0.0001, ns: not significant)

In accordance with the experimental design presented in Fig. 7a, three weeks after booster immunization, serum samples were collected and used to conduct hemagglutination inhibition (HI), neuraminidase inhibition (NI), and virus neutralization (VN) assays. Three types of antigens were used for the serological assays: the 22W_KY vaccine strain and the SNU50-5 and PR8 challange strain viruses.

A HI response against any of the three viral antigens was undetectable for all vaccine groups (Fig. 7b). In the NI assays, all vaccine groups had significantly higher NI titers against the 22W_KY and SNU50-5 antigens than the negative control group did, with no significant differences observed among the vaccine groups (Fig. 7c). None of the vaccine groups had detectable NI titers against the PR8 antigen. VN assay revealed that all vaccine groups had significantly higher neutralization titers against 22W_KY than did the negative control group, but no significant differences were found among the vaccine groups (Fig. 7d). Compared with the negative control group, all the vaccine groups had detectable neutralization activity against the SNU50-5 antigen, but there were no significant differences among all the groups. None of the vaccine groups exhibited neutralization activity against the PR8 antigen.

### Robust IgG and IgA antibody responses in the BEI-inactivated 22W_KY group

**Fig. 8:**
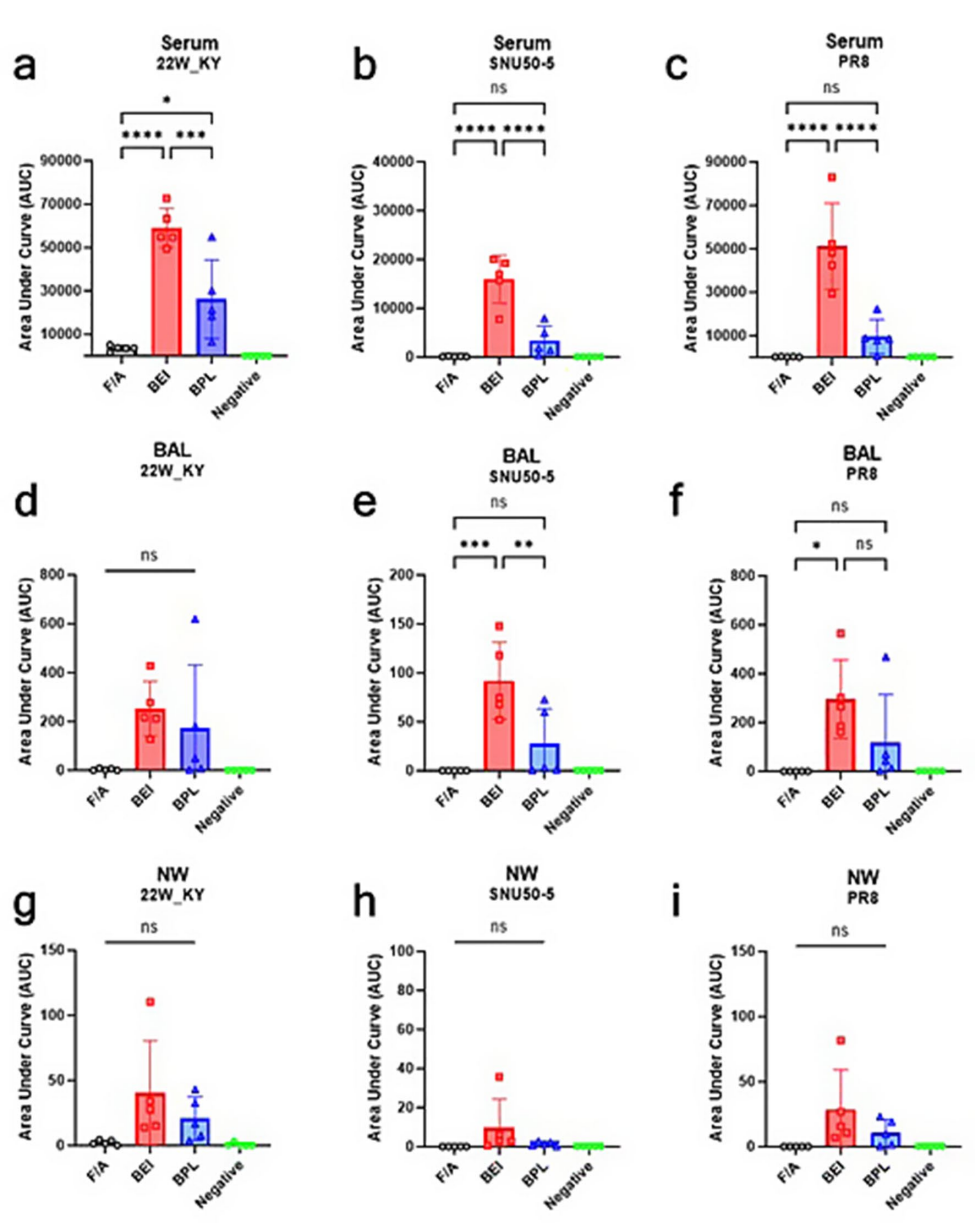
Intranasal administration of the BEI-inactivated 22W_KY virus induces the robust serum IgG responses against three distinct antigens in mice. Serum, NW, and BAL samples were collected three weeks after booster immunization to evaluate antigen-specific IgG responses. The IgG responses against the homologous vaccine strain, (**a**,** d**,** g**) 22W_KY, heterologous antigen, (**b**,** e**,** h**) SNU50-5 and heterosubtypic antigen (**c**,** f**,** i**) PR8, were measured by antigen-specific ELISA (*n* = 5). The IgG responses elicited by vaccines inactivated with F/A, BEI, and BPL were quantified and are presented as the area under the curve (AUC). All the data are presented as the mean ± SD, and statistical significance was analyzed using one-way ANOVA with Tukey’s multiple comparisons test (*p < 0.05, **p < 0.01, ***p < 0.001, ****p < 0.0001)

Compared with F/A_22W_KY and BPL_22W_KY, BEI_22W_KY elicited the strongest serum-IgG antibody responses across all antigens tested, with significantly greater responses for the 22W_KY, SNU50-5, and PR8 antigens (Fig. 8a, b, c). In bronchoalveolar lavage (BAL) fluid, there were no significant differences among the three groups in terms of response to homologous antigens (22W_KY) (Fig. 8d). However, BEI_22W_KY elicited a significantly stronger BAL-IgG response against both SNU50-5 and PR8 than F/A_22W_KY did (Fig. 8e, f). No significant differences in nasal wash (NW) IgG responses were observed among the three inactivation groups (Fig. 8g, h, i).

**Fig. 9:**
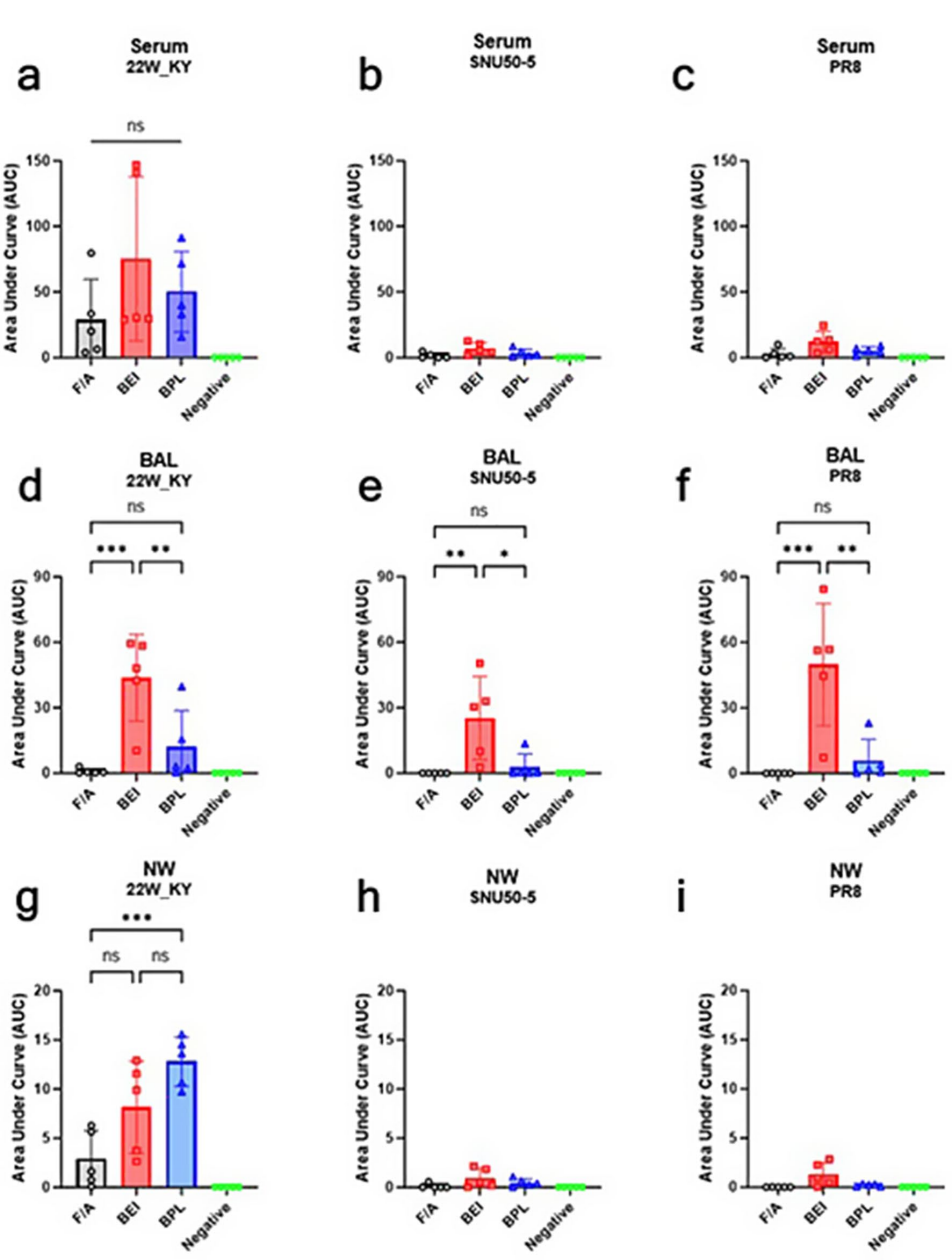
Intranasal administration of the BEI-inactivated 22W_KY virus induces the robust IgA responses in bronchoalveolar lavage (BAL) fluid against three distinct antigens in mice. The IgA responses against the homologous vaccine strain, (**a**,** d**,** g**) 22W_KY, heterologous antigen, (**b**,** e**,** h**) SNU50-5 and heterosubtypic antigen (**c**,** f**,** i**) PR8, were measured by antigen-specific ELISA (*n* = 5). The IgA responses elicited by viruses inactivated with F/A, BEI, and BPL were quantified and presented as the area under the curve (AUC). All the data are presented as the mean ± SD, and statistical significance was analyzed using one-way ANOVA with Tukey’s multiple comparisons test (*p < 0.05, **p < 0.01, ***p < 0.001, ****p < 0.0001)

Compared with the negative control, all the vaccines induced higher serum IgA responses to 22W_KY antigen, but no significant differences were observed among the three groups (Fig. 9a). With respect to the SNU50-5 and PR8 antigens, all vaccines induced minimal to no measurable serum IgA responses (Fig. 9b, c). In BAL fluid, F/A_22W_KY group induced minimal to no measurable IgA responses to the three antigens, whereas BEI_22W_KY induced significantly greater IgA responses than F/A_22W_KY and BPL_22W_KY did (Fig. 9d, e, f). Compared with those induced by F/A_22W_KY and BPL_22W_KY, the NW-IgA responses induced by BEI_22W_KY were not significantly different against the homologous antigen (Fig. 9g). None of the vaccines generated substantial NW-IgA responses to heterologous antigens (Fig. 9h, i). Overall, BEI_22W_KY elicited significantly greater serum-IgG and BAL-IgA responses than F/A_22W_KY or BPL_22W_KY did, indicating that intranasal administration of BEI-inactivated antigens can effectively induce both strong systemic IgG responses and robust mucosal immune responses in the lungs.

### Antigen-specific T-cell immune responses induced by intranasal administration of inactivated 22W_KY

**Fig. 10:**
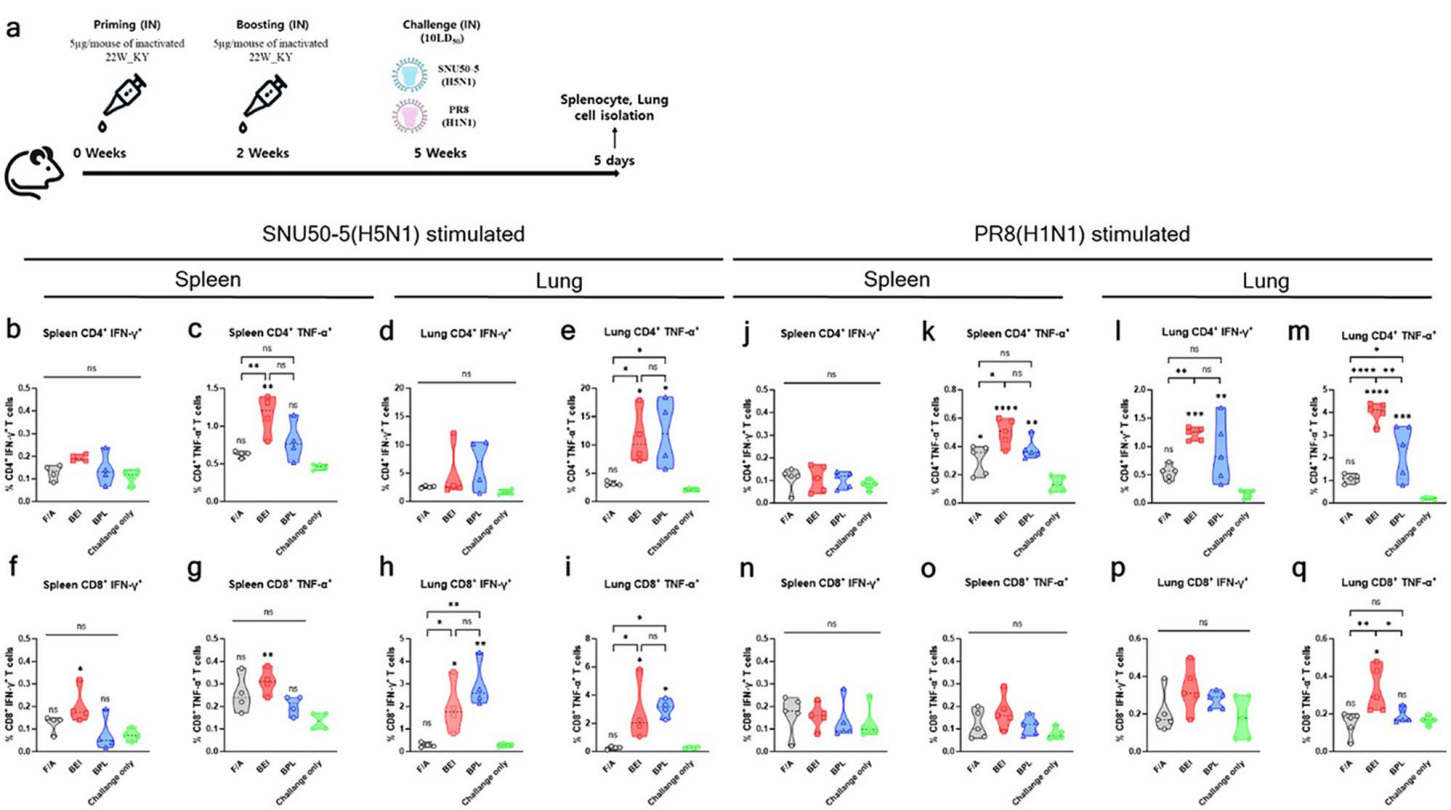
BEI-inactivated 22W_KY induces pronounced TNF-α + T-cell responses, with stronger immune activation in the lungs than in the spleen. (**a**) To assess T-cell responses induced by different vaccine inactivation methods, six-week-old BALB/c mice (*n* = 4–5) were intranasally immunized with F/A-, BEI-, or BPL-inactivated 22W_KY. Three weeks after booster vaccination, the mice were challenged with 10 LD_50_ of either the SNU50-5 or the PR8 virus. Five days after the challenge, splenocytes and immune cells isolated from the lungs were restimulated in vitro with the respective challenge viruses to evaluate antigen-specific cytokine-secreting T-cell populations. The frequencies of (**b**,** d**,** j**,** l**) IFN-γ-producing and (**c**,** e**,** k**,** m**) TNF-α-producing CD4 + T cells and the frequencies of (**f**,** h**,** n**,** p**) IFN-γ-producing and (**g**,** i**,** o**,** q**) TNF-α-producing CD8 + T cells were measured. Fluorescence minus one (FMO) controls were used to gate IFN-γ and TNF-α positive populations. All the data are presented as the mean ± SD, and statistical significance was analyzed using one-way ANOVA with Tukey’s multiple comparisons test. Significant differences are denoted by asterisks, with asterisks displayed directly above the violin plots indicating statistical significance compared with the negative control (challenge-only group). (*p < 0.05, **p < 0.01, ***p < 0.001, ****p < 0.0001, ns: not significant)

To evaluate the antigen-specific T-cell responses elicited by different inactivation methods, five days after challenge with either SNU50-5 or PR8 virus, splenocytes and immune cells isolated from the lung were restimulated with the respective challenge viruses and analyzed via an antigen-specific intracellular cytokine staining assay (Fig. 10a). The gating strategy used to identify T-cell subsets is shown in Supplementary Fig. 6. Representative flow images of the T-cell response under SNU50-5 or PR8 stimulation are shown in Supplementary Fig. 7.

A significant increase in the frequencies of CD4 + TNF-α+, CD8 + IFN-γ+, and CD8 + TNF-α + T cells in the lung upon restimulation with SNU50-5 were observed in the BEI_22W_KY and BPL_22W_KY groups compared with the control group, with the F/A_22W_KY group showing frequencies comparable to those of the control group (Fig. 10e, h, i). Upon PR8 antigen restimulation, significantly greater frequencies of CD4 + IFN-γ + cells were observed in the lungs of both the BEI_22W_KY and BPL_22W_KY groups than the control group, with the levels in the F/A_22W_KY group being comparable to those in the control group (Fig. 10l). Notably, a significant increase in the CD4 + TNF-α + and CD8 + TNF-α + T-cells responses in the lung upon restimulation with PR8 was observed in the BEI_22W_KY group compared with the F/A_22W_KY and BPL_22W_KY group (Fig. 10m, q). The highest frequency of TNF-α-producing CD4 + T-cell responses in the splenocyte upon restimulation with both SNU50-5 and PR8 was observed in the BEI_22W_KY group (Fig. 10c, k). However, compared with the T-cell responses observed in the lung, remarkably lower frequency of cytokine-secreting T cells upon restimulation was observed in the splenocyte.

Overall, intranasal administration of the F/A_22W_KY did not significantly increase T-cell responses in lymphocytes from either the spleen or lungs. Both BEI_22W_KY and BPL_22W_KY elicited comparable increases in SNU50-5-specific cellular immunity. Notably, BEI_22W_KY induced the most pronounced increase in TNF-α + T-cell responses among PR8-specific T-cell responses, particularly in the lungs rather than in the spleen.

## Discussion

Clade 2.3.4.4b H5N1 has rapidly evolved into a significant global threat, infecting a diverse range of hosts, including poultry, birds of prey, carnivores, marine mammals, and humans [6, 7, 13, 46, 47]. Given these risks, it is essential to prioritize the development of H5N1 vaccines not only for poultry but also for mammals, addressing cross-species transmission and the significant implications for public health. Additionally, to prepare for unexpected variants and the potential emergence of pandemic influenza, it is crucial to develop vaccines capable of providing cross-protection against diverse strains.

Evolutionary analysis revealed that clade 2.3.4.4b H5N1 isolates only in East Asia diverged into two distinct patterns during the early phase of emergence, on the basis of HA amino acid sequences: the 21 W and 22 W patterns (Fig. 1 and Supplementary Fig. 1). This divergence is characterized by specific mutations at residues 90, 93, 125, and 193 of HA. Among the four specific mutations analyzed, the HA-R90K mutation significantly increased the heat stability of the 22W_MVV virus (Fig. 2). Notably, this finding is a novel, as the R90K mutation has not been identified as a factor influencing the stability of HA in any previous studies. The stability of HA is influenced primarily by electrostatic interactions at its structural interface, such as hydrogen bonds, salt bridges, and van der Waals forces [48]. The key residues contributing to this stability are primarily located in three critical regions: the fusion peptide region, the HA1–HA1 interface, and the HA1–HA2 interface [49, 50]. Residue 90 of HA, located within the vestigial esterase domain (VED) beneath the receptor-binding domain (RBD) and distant from other HA monomers, likely influences protein stability by modulating interactions at the HA1–HA2 interface (Supplementary Fig. 5). The interaction at the HA1–HA2 interface is predominantly formed between the B-loop of HA2 and the VED or fusion subdomain of HA1, involving residues from HA1 (positions 85–90, 104–115, and 265–270) and HA2 (positions 64–72) [48]. One notable example is a tetrad salt bridge between E89 and R109 of HA1 and E67 and R269 of HA2, which has been reported to significantly influence protein stability [51]. Although residue 90 is distant from other monomers, its proximity to residue 89 suggests a potential role in modulating interactions between the VED and the B-loop. However, since both arginine (R) and lysine (K) are positively charged basic amino acids, it is unlikely that the structural stabilization conferred by the R90K mutation results from changes in salt bridge formation but may instead affect the interaction between the B-loop and the VED domain through an alternative mechanism. Although this study could not elucidate the exact mechanism underlying the increased structural stability associated with the R90K mutation, further studies are needed to clarify how this mutation increases HA stability. On the other hand, Y110 is known to form a hydrogen bond with N413 in the Helix C region of the HA2 of the adjacent monomer, contributing to the stabilization of the trimeric protein structure [44, 52]. This property was also applicable to the HA protein of the our clade 2.3.4.4b H5N1 vaccine strain. Notably, the addition of the H110Y mutation further synergized with R90K, resulting in maintained HA activity for both live and inactivated viruses even after four hours at 52 °C (Fig. 2b and c). The increased stability of this vaccine strain offers significant advantages, both as an intranasal vaccine—enabling prolonged retention within respiratory mucosal barriers—and as a WIV vaccine, ensuring effective storage and transport in resource-limited settings without the need for a cold chain [53].

The N193D mutation significantly reduced the receptor-binding affinity for 2,3-SA and altered the antigenicity of the HA RBD (Supplementary Fig. 2 and Table 2). Residue 193 in HA is located within the 190-helix of the RBD, a region known to play critical role in modulating receptor-binding affinity [54]. This residue is also a key component of antigenic site B, which serves as an immunodominant epitope within clade 2.3.4.4b H5 HPAIVs (Supplementary Fig. 5) [55]. Furthermore, viruses with high receptor-binding affinity can evade antisera in HI assays by binding to cells more efficiently [56]. Based on these previous findings, the lower HI titers of the antisera induced by the the D193-containing vaccine strain (21W_sera) against the 22W_MVV antigen could be attributed to the inability of 21W_sera to sufficiently recognize the RBD epitope containing N193 residue. This insufficient recognition likely allowed the 22W_MVV antigen, with high receptor-binding affinity, to bind to RBCs effectively (Table 2). In contrast, the antiserum raised against the N193-containing vaccine strain (22W_sera) effectively bound to the RBD epitope at N193 residue, successfully inhibiting HA even in viruses with high receptor-binding affinity. These findings provide a potential explanation for the restricted circulation limited to East Asia and the rapid decline after 2022 of clade 2.3.4.4b H5N1 viruses with the 21 W HA pattern (Fig. 1 and Supplementary Fig. 1). Moreover, they underscore the importance of RBD epitopes as a critical consideration in vaccine design for viruses with high receptor-binding affinity. Considering the global dominance of clade 2.3.4.4b H5N1 viruses with the N193 residue, the selection of vaccine strains that include N193 is essential for effective immunization strategies.

The development of vaccine strains with high antigen yields and low mammalian infectivity is crucial for the efficient and safe production of WIV vaccines, particularly given the pandemic potential of clade 2.3.4.4b H5N1 viruses [14]. By incorporating the 310-MVV PB2 gene, we successfully addressed both challenges. Using infectivity assays and western blot analysis, we found that the 310-MVV PB2 gene is highly compatible with the HA and NA of clade 2.3.4.4b H5N1 vaccine strains in ECEs. Interestingly, although the NP band intensities were similar among the three groups (22W_PR8, 22W_MVV, and 22W_KY), the 22W_PR8 HA1 band intensity was significantly lower (Fig. 3a and Supplementary Fig. 3). Given that most influenza virus particles contain exactly one set of eight RNP complexes, similar NP levels imply that a comparable number of total virions was produced in each group [57]. The weaker HA1 band observed for 22W_PR8 only suggests that HA expression or membrane trafficking efficiency might be compromised in the ECE environment for this strain. Conversely, viruses carrying the avian-derived PB2 gene 310-MVV, such as 22W_MVV and 22W_KY, may exhibit greater adaptation to ECEs, resulting in more efficient HA protein expression or surface trafficking than the conventional PR8-based PB2. A recent study employing H1N1 vaccine antigens reported that modifying PB1 changes the ratio of HA and NA expression levels and can increase the NA content [58]. This finding underscores that polymerase variants could influence the expression and packaging efficiency of surface glycoproteins (i.e., HA or NA). Moreover, vaccine strains containing the 310-MVV PB2 gene had significantly reduced replication efficiency in mammalian (MDCK) and human respiratory (Calu-3) cell lines, as well as in the lung tissues of BALB/c mice, demonstrating that the 310-MVV PB2 gene has significant advantages not only in terms of antigen yield but also in terms of biosafety (Fig. 4). Therefore, the improved 22W_KY strain was ultimately selected as the intranasal WIV vaccine strain due to its superior stability, cost-effectiveness, and enhanced biosafety.

Interestingly, measurable HI activity against any of the tested antigens was not observed in the serum samples from any of the vaccine groups (Fig. 7b). This finding could suggest that none of the vaccines induced sufficient HI antibodies. Alternatively, assay-related issues, such as nonspecific agglutination by serum natural agglutinins or the lower sensitivity of chicken RBCs than that of horse RBCs for detecting HI responses against avian H5Nx viruses, may have influenced these results [59–61]. However, we experimentally confirmed that nonspecific agglutination did not occur in the serum samples in the absence of virus. Additionally, in a separate experiment, mice intramuscularly immunized with an oil emulsion 22W_KY vaccine presented high HI titers (2^6^–2^7^) when the same chicken RBC-based assay was used, indicating that the assay sensitivity was generally adequate (data not shown). Although some low-level HI responses might have been detected in horse RBCs, the consistent absence of detectable HI titers across all intranasally vaccinated groups strongly indicates that intranasal administration of these vaccine strains was ineffective in eliciting robust HI antibody responses.

In contrast, considerable NI and VN antibody responses against the 22W_KY and SNU50-5 antigens, were observed in all vaccine groups, while responses against PR8 were undetectable (Fig. 7c, d). This finding may be attributable to the high amino acid sequence homology of the NAs between SNU50-5 and the vaccine strain (97.2%), compared with the lower homology of PR8 NA (83.5%), justifying why all vaccine groups showed better protection against SNU50-5 than PR8 (Supplementary Table 2). However, a clear limitation of our NI assay was the use of whole virions as antigens. In particular, all detectable NI responses were observed only when antigens with the same HA subtype as the vaccine strain were used. Thus, we cannot exclude the possibility that antibodies binding to the HA protein, rather than purely NA-specific antibodies, influenced these results [62]. Future studies should employ NI assays using viral antigens with different HA subtypes from the vaccine strain or conduct ELISAs with purified HA and NA proteins to more precisely differentiate HA- and NA-specific antibody responses.

Interestingly, despite the lack of significant differences in HI, NI, and VN responses among the vaccine groups, BEI_22W_KY elicited the highest serum IgG responses against all the tested antigens (Fig. 8a, b, c). Given the lack of significant differences in HI and NI responses, the notably high serum IgG levels observed against PR8 are unlikely to target HA or NA. Instead, these antibodies may recognize conserved regions such as M2e or internal proteins such as NP and M1. While M2e is expressed on the surface of influenza viruses, its low copy number makes it a less likely target for the robust IgG response observed [63]. In contrast, the high abundance of NP and M1 proteins within influenza virions and the observation that BEI_22W_KY elicited greater IgG responses to 22W_KY and PR8 antigens, which share identical internal genes, than to the SNU50-5 antigen suggest that NP and M1 proteins are the primary targets of the serum IgG response. Although nonneutralizing antibodies against NP and M1 proteins are generally not sufficient to mediate antibody-dependent cellular cytotoxicity (ADCC) or to directly inhibit virus replication, studies indicate that these antibodies, when complexed with NP and M1 immune complexes, can activate natural killer (NK) cells [64–66]. This activation can stimulate innate immune cells to secrete cytokines, contributing to an antiviral environment during the early stages of infection. Thus, while these antibodies may not prevent virus entry or replication directly, they likely play a role in modulating the immune environment to limit virus spread and disease progression. Further research is needed to identify the specific viral proteins or epitopes responsible for the increased serum IgG response, which will provide valuable insights into the underlying mechanisms and guide the design of more effective vaccines.

Compared with other vaccine, with BEI_22W_KY elicited significantly stronger mucosal IgA antibody responses in BAL fluid (Fig. 9). This finding indicates that BEI_22W_KY effectively penetrates the respiratory mucosal barrier, activating the mucosal immune system. IgA produced in the respiratory mucosa serves as the first line of defense by neutralizing the virus at the site of infection before it can replicate, and IgA has broader reactivity against diverse influenza variants than does IgG [26]. These findings highlight the potential of BEI_22W_KY as an effective intranasal vaccine. In addition to humoral responses, T-cell immunity is less affected by virus mutations and plays a critical role in protection, including reducing viral shedding and alleviating symptoms [67]. Therefore, eliciting a strong T-cell response is crucial for developing vaccines that provide broad protection against multiple variants and achieve sterile immunity. In our study, T-cell responses were consistently more pronounced in the lungs than in the spleen across all WIV groups, underscoring the pivotal role of localized mucosal immunity in determining the efficacy of WIV vaccines. Upon restimulation with each challenge virus antigen, BEI_22W_KY strongly induced both CD4 + and CD8 + TNF-α + T-cell responses in the lungs (Fig. 10). TNF-α plays a critical role in promoting inflammatory responses, improving antigen-presenting cell (APC) functions, and regulating overall immune responses [68, 69]. Although no significant differences in TNF-α responses in the lungs after SNU50-5 virus challenge were observed after BEI_22W_KY and BPL_22W_KY vaccination, BEI_22W_KY elicited a markedly stronger response to challenge with the PR8 virus, as it shares identical internal genes. In addition to the IgG response analysis, these findings further support the hypothesis that intranasal administration of BEI_22W_KY effectively induce immune responses that target internal proteins (e.g., NP or M1), potentially contributing to its complete prevention of weight loss and virus shedding in response to a lethal PR8 virus challenge. Overall, these results highlight the high potential of BEI_22W_KY to provide broad protection by eliciting immune responses against not only surface proteins but also internal viral proteins.

Notably, unlike inactivated 22W_KY, the thermal stability of inactivated 22W_MVV varied depending on the method of inactivation. Live 22W_MVV exhibited very low thermal stability, whereas F/A-inactivated 22W_MVV demonstrated remarkably high thermal stability. BPL-inactivated 22W_MVV showed intermediate stability, while BEI-inactivated 22W_MVV had very low stability similar to live 22W_MVV. Conversely, viral internalization efficiency into cells and the protective efficacy of intranasal vaccination displayed the opposite ranking (BEI > BPL > F/A). These contrasting results can be interpreted by the differing impacts each inactivation method has on the structural flexibility of the HA protein. F/A treatment forms methylene cross-links between viral surface proteins, effectively “stapling” the HA structure [70]. As a result, F/A-treated HA acquires increased structural resistance to heat-induced denaturation and dissociation. However, this rigidity restricts the conformational changes required for HA-mediated viral entry into the cytoplasm, thereby reducing antigen uptake efficiency and overall immunogenicity [71]. BPL-inactivated viruses showed intermediate thermal stability and immunogenicity, likely because BPL primarily alkylates viral nucleic acids but also induces partial acetylation and some protein cross-linking during the inactivation process [70]. In contrast, BEI treatment, which primarily inactivates viruses by alkylating the viral genome, induces minimal protein cross-linking, thus preserving HA structural flexibility similar to that of live viruses [72, 73]. The low thermal stability observed in BEI-inactivated 22W_MVV, comparable to that of the live virus, suggests retention of natural structural flexibility. Consequently, this flexibility allows more efficient viral entry into host cells and better recognition by the immune system, leading to the strongest immunogenic response. Therefore, thermally stabilized strain 22W_KY, when inactivated by BEI, may achieve an optimal balance between stability and immunogenicity by maintaining high thermal durability and preserving antigen structural flexibility.

This study has several limitations, including the use of only a single animal model and the evaluation of protective efficacy against only two challenge virus strains. Further research should focus on assessing the efficacy of intranasal WIV vaccines in other animal models, such as chickens or other mammalian species, and evaluating their protective potential against a broader range of challenge strains.

## Conclusions

In conclusion, we identified the HA-R90K mutation as critical for enhancing the structural stability of our clade 2.3.4.4b H5N1 vaccine strain. We also introduced PB2 modifications (310-MVV PB2), which not only increased antigen production in embryonated chicken eggs but also reduced mammalian infectivity, thereby improving both the productivity and biosafety of our vaccine compared with the conventional PR8 backbone. Furthermore, upon BEI inactivation, the optimized vaccine strain (22W_KY) efficiently entered respiratory epithelial cells in vitro and provided complete protection against heterologous H5N1 challenge (SNU50-5) and significant cross-protection against heterosubtypic H1N1 challenge (PR8) in mice. Notably, intranasal administration of BEI-inactivated 22W_KY (BEI_22W_KY) elicited robust IgG and mucosal IgA responses, along with increased TNF-α + T-cell responses in the respiratory tract, highlighting the potential of this intranasal WIV vaccine to elicit both systemic and local immunity. Taken together, our findings underscore the potential of BEI_22W_KY as a productive, safe, stable, and broadly protective intranasal influenza vaccine candidate, offering an effective strategy to combat the ongoing threat of clade 2.3.4.4b H5N1 and potentially other emerging influenza strains.

## Electronic supplementary material

Below is the link to the electronic supplementary material.

Supplementary Material 1

## Data Availability

All data generated or analyzed during this study are included in this manuscript or supplementary information. All relevant data are available upon reasonable request from the corresponding author.

## Abbreviations

ADCC: Antibody-Dependent Cellular Cytotoxicity
APC: Antigen-Presenting Cell
BEAST: Bayesian Evolutionary Analysis Sampling Trees
BEI: Binary Ethylenimine
BAL: Bronchoalveolar Lavage
BPL: β-Propiolactone
CI: Confidence Interval
ELISA: Enzyme-Linked Immunosorbent Assay
ELLA: Enzyme-Linked Lectin Assay
ECEs: Embryonated Chicken Eggs
EID_50_: 50% Egg Infectious Dose
F/A: Formaldehyde
GMT: Geometric Mean Titer
GISAID: Global Initiative for Sharing All Influenza Data
Gs/Gd-lineage: Goose/Guangdong lineage
HA: Hemagglutinin
HI: Hemagglutination Inhibition
HPAIV: Highly Pathogenic Avian Influenza Virus
IFN-γ: Interferon-Gamma
LAIV: Live Attenuated Influenza Virus
M1: Matrix Protein 1
M2e: Extracellular Domain of Matrix Protein 2
MFI: Mean Fluorescence Intensity
MDCK: Madin-Darby Canine Kidney
MOI: Multiplicity of Infection
NA: Neuraminidase
NI: Neuraminidase Inhibition
NK: Natural Killer
NP: Nucleoprotein
NT: Nasal Turbinate
NW: Nasal Wash
OD: Optical Density
PB1, PB2, PA: Polymerase Basic 1 & 2, Polymerase Acidic
RBC: Red Blood Cell
RBD: Receptor Binding Domain
RNP: Ribonucleoprotein
SA: Sialic Acid
SD: Standard Deviation
SPF: Specific Pathogen Free
sIgA: Secretory Immunoglobulin A
sIgG: Secretory Immunoglobulin G
TCID_50_: 50% Tissue Culture Infectious Dose
TNF-α: Tumor Necrosis Factor-alpha
VED: Vestigial Esterase Domain
VN: Virus Neutralization
WIV: Whole Inactivated Virus
