# Supplementary material for: Intranasally administered whole virion inactivated vaccine against clade 2.3.4.4b H5N1 influenza virus with optimized antigen and increased cross-protection
Source: Virol J. 2025 May 5;22:131. doi: 10.1186/s12985-025-02760-4 (PMC12051338; doi:10.1186/s12985-025-02760-4)
Supplement: Supplementary file 1 — Supplementary Material 1 [file 12985_2025_2760_MOESM1_ESM.pdf]

Supplementary information

22W HA and 22W NA sequence

>22W HA (Accession number : PQ554516)

MENIVLLLAIVSLVKSDQICIGYHANNSTEQVDTIMEKNVTVTHAQDILEKTHNGKLCDLNGV
KPLILKDCSVAGWLLGNPMCDEFIRVPEWSYIVERANPANDLCYPGSLNDYEELKHLLSRINH
FEKILIIPKSSWPNHETSLGVSAACPYQGAPSFFRNVVWLIKKNDAYPTIKISYNNTNREDLLIL
WGIHHSNNAEEQTNLYKNPTTYISVGTSTLNQRLVPKIATRSQVNGQRGRMDFFWTILKPDD
AIHFESNGNFIAPEYAYKIVKKGDSTIMKSGVEYGHCNTKCQTPVGAINSSMPFHNIHPLTIGE
CPKYVKSNKLVLATGLRNSPLASGRGLFGAIAGFIEGGWQGMVDGWYGYHHSNEQGSGYAA
DKESTQKAIDGVTNKVNSIIDKMNTQFEAVGREFNNLERRIENLNKKMEDGFLDVWTYNAEL
LVLMENERTLDFHDSNVKNLYDKVRLQLRDNAKELGNGCFEFYHKCDNECMESVRNGTYD
YPQYSEEARLKREEISGVKLESIGTYQILSIYSTAASSLALAIMIAGLSLWMCSNGSLQCRICI

>22W NA (Accession number : PQ554517)

MNPNQRIITTGSICMVIGIVSLMLQIGNIISIWVSHSIQTGNQYQPEPCNQSIITYENNTWVNQT
YVNISNTNFLAEQAVTSVTLAGNSSLCPISGWAIYSKDNGIRIGSKGDVFVIREPFISCSHLECRT
FFLTQGALLNDKHSNGTVKDRSPYRTLMSCPVGEAPSPYNSRFESVAWSASACHDGISWLTIG
ISGPDNGAVAVLKYNGIITDTIKSWRNNILRTQESECACVNGSCFTVMTDGPSNGQASYKIFKI
EKGKVVKSVELNAPNYHYEECSCYPDAGEIMCVCRDNWHGSNRPWVSFNQNLEYQIGYICS
GVFGDNPRPNDGTGSCSPMSSNGAYGVKGFSFKYGNGVWIGRTKSTSSRSGFEMIWDPNGW
TETDSSFSVKQDIVAITDWSGYSGTFVQHPELTGLDCMRPCFWVELIRGRPKENTIWTSGSSIS
FCGVNSDTVGWSWPDGAELPFTIDK

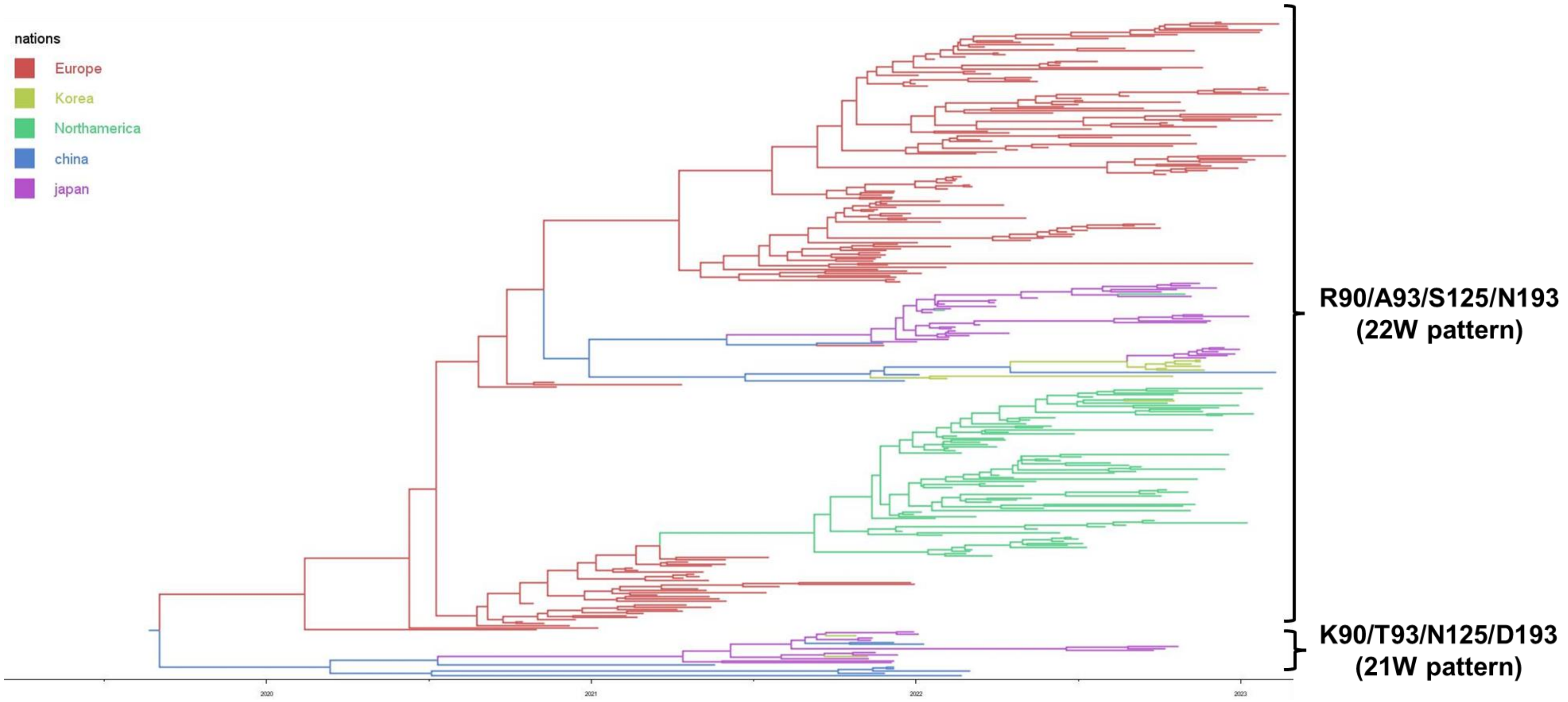

Supplementary Fig. 1. Time-scaled phylogenetic and genetic analysis of clade 2.3.4.4b H5N1 hemagglutinin amino acid sequences isolated from East Asia, North America, and Europe. Time-scaled phylogenetic tree showing the evolutionary relationships of HA sequences of clade 2.3.4.4b H5N1 viruses collected between October 2021 and February 2023. From the 1,633 European sequences and 1,292 North American sequences available in GISAID, 167 European sequences and 83 North American sequences were randomly selected and used for phylogenetic analysis alongside East Asian sequences. Color coding represents the regions where the samples were collected, as indicated by the legend on the left.

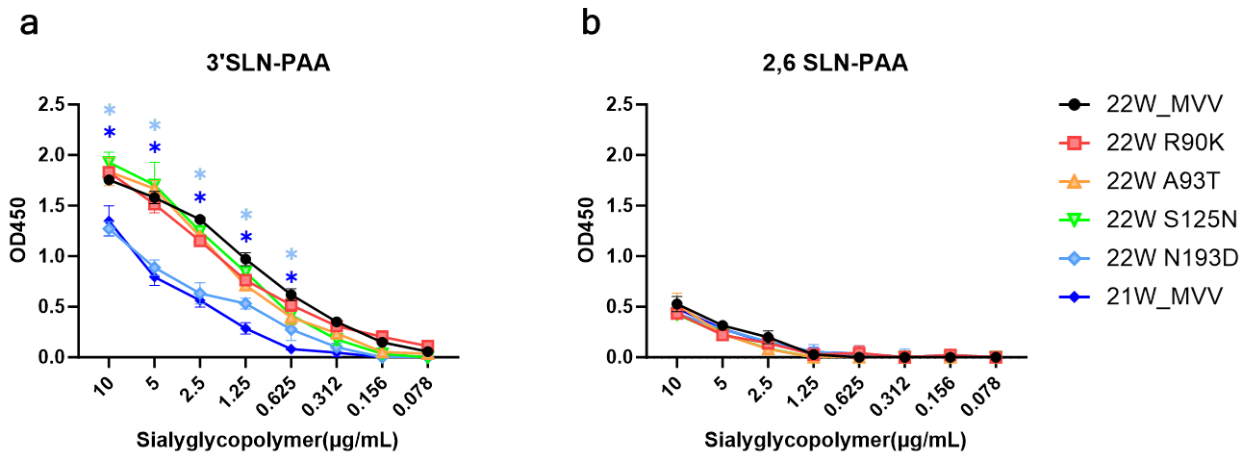

Supplementary Fig. 2. Solid-phase receptor bindng assay of recombinant H5N1 viruses. The binding of each recombinant H5N1 virus to sialylglycopolymers—**a)** α2,3-linked (3'SLN-PAA) and **b)** α2,6-linked (6'SLN-PAA)—was evaluated using a solid-phase binding assay. Absorbance data are presented as mean ± standard deviation (SD) from triplicate experiments. Statistical significance between 22W H5N1 and other recombinant H5N1 viruses was analyzed using two-way ANOVA, followed by Dunnett's multiple comparisons test. Significant difference between 22W_MVV and 21W_MVV is indicated by blue asterisks, while difference between 22W_MVV and 22W N193D is marked by light blue asterisks (*$p < 0.0001$).

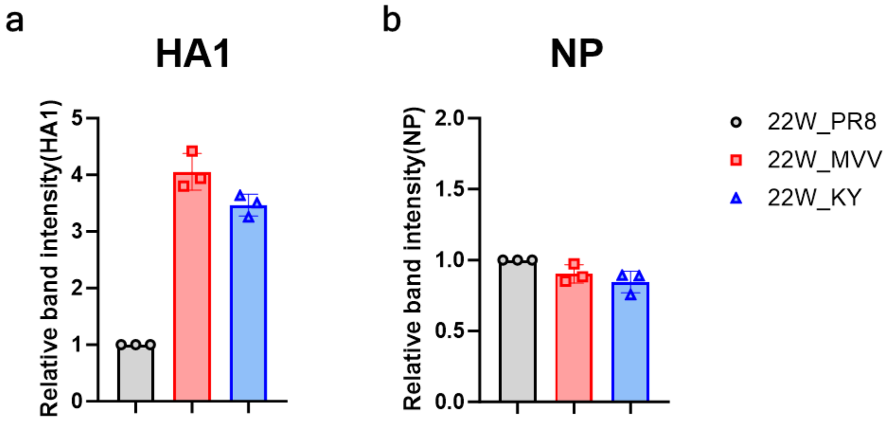

Supplementary Fig. 3. The relative band intensity of HA1 and NP for each recombinant H5N1 vaccine strain. The intensities of western blot bands for each recombinant H5N1 vaccine strain were measured using ImageJ software. The relative band intensities of the **a)** HA1 band and the **b)** NP band are plotted in the graph. The intensity of the 22W_PR8 band was set to 1, and the relative intensities of the 22W_MVV and 22W_KY bands were calculated and displayed in a graph.

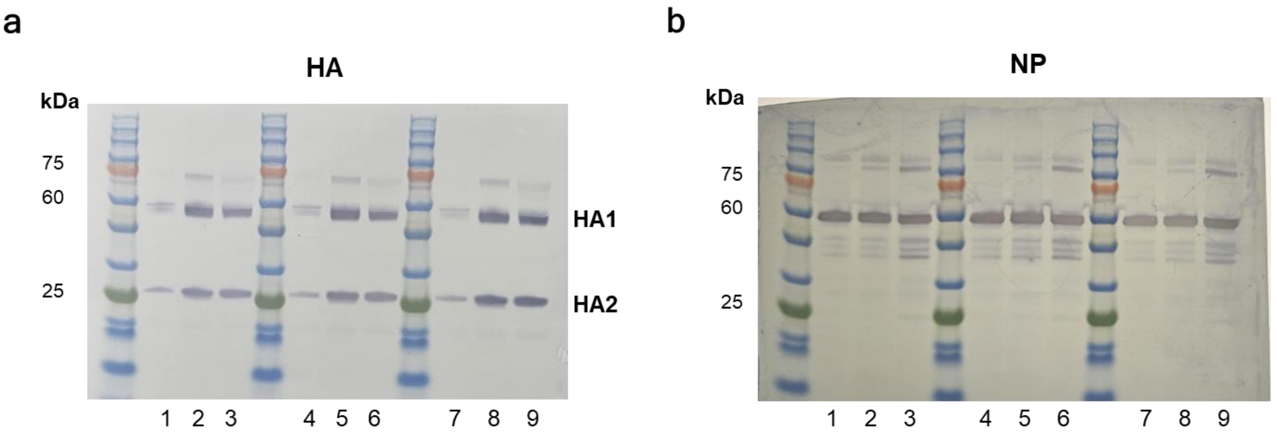

1 : 22W_PR8 - Replicate1
2 : 22W_MVV - Replicate1
3 : 22W_KY - Replicate1
4 : 22W_PR8 - Replicate2
5 : 22W_MVV - Replicate2
6 : 22W_KY - Replicate2
7 : 22W_PR8 - Replicate3
8 : 22W_MVV - Replicate3
9 : 22W_KY - Replicate3

Supplementary Fig. 4. Un-cropped and unprocessed western blot image to compare of the a) HA and b) NP antigen levels of recombinant H5N1 vaccine strains.

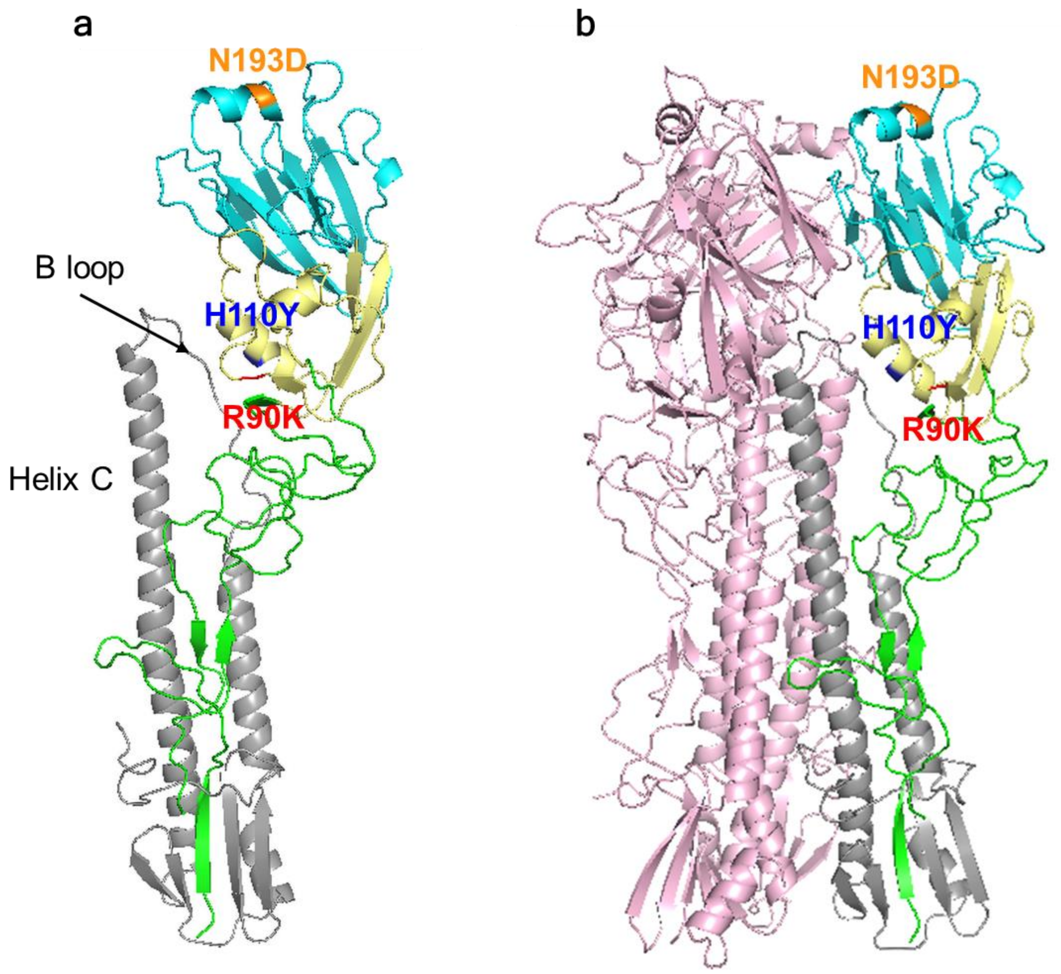

Supplementary Fig. 5. The HA structure of the 22W_KY strain. Using the AlphaFold 3 model, we predicted the structures of the H5 protein derived from 22W_KY, analyzing it as either **a)** monomer or **b)** trimer. The predicted structures were visualized with PyMOL v4.6.0. The HA protein structure is annotated as follows: the fusion domain is highlighted in green, the vestigial esterase domain (VED) in yellow, and the receptor-binding domain (RBD) in cyan, while the HA2 region is shown in gray. Key mutations are also color-coded, with R90K in red, H110Y in blue, and N193D in orange.

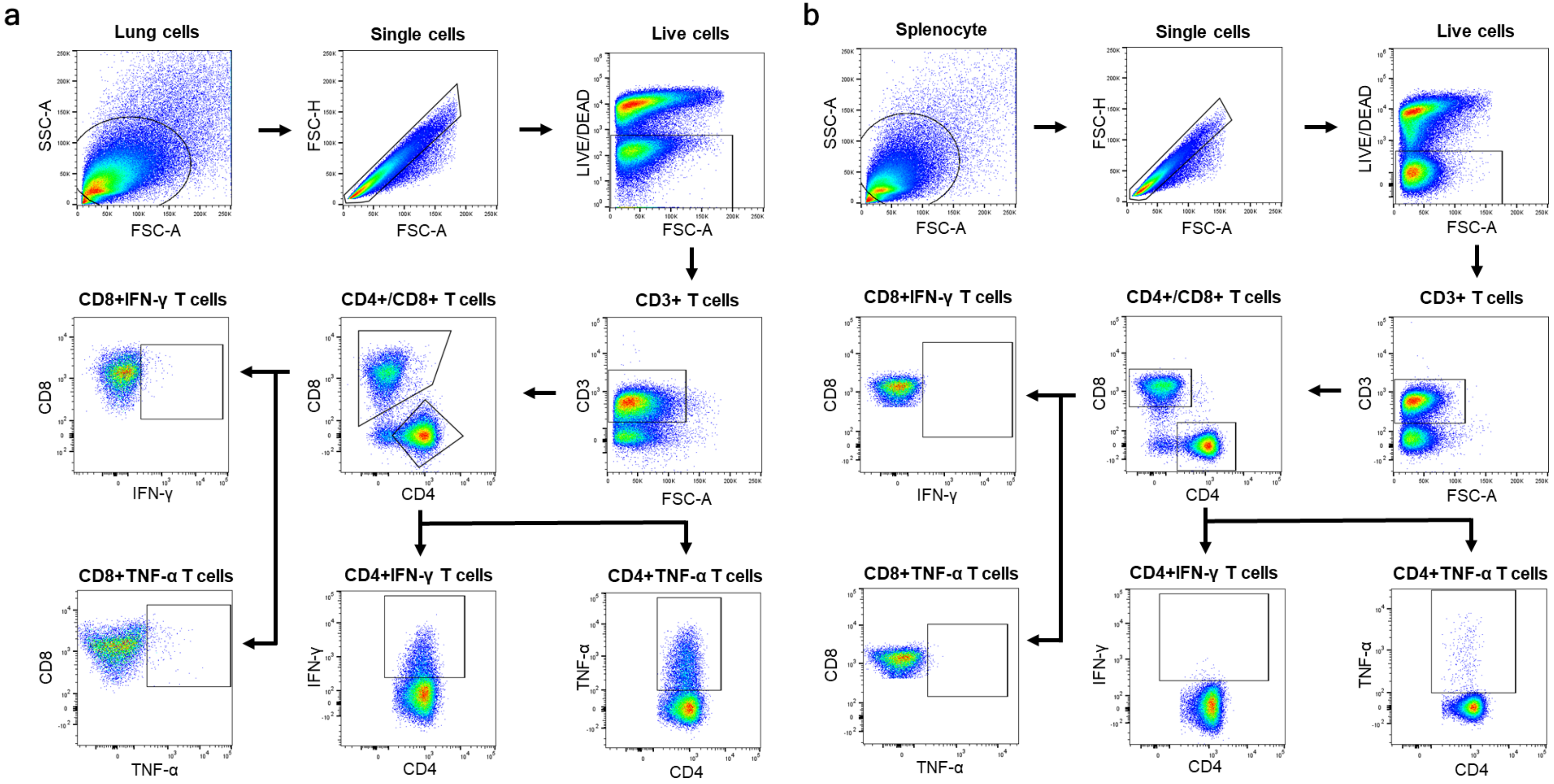

Supplementary Fig. 6. Schematics of the gating strategy for the analysis of T cell subsets in the a) lung or b) spleen by flow cytometry.

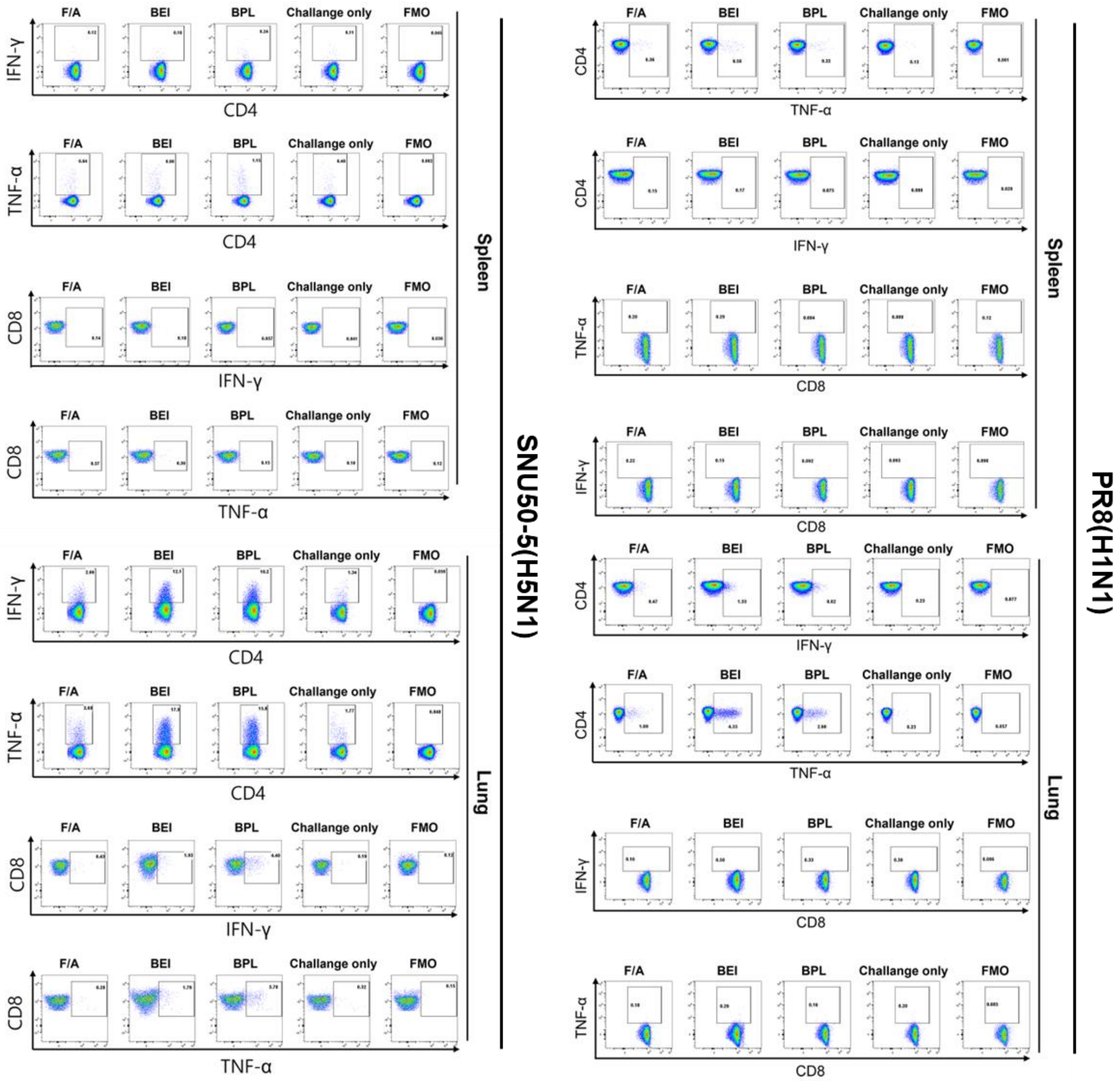

Supplementary Fig. 7. Representative flow cytometry images of T cells response in spleen and lung under SNU50-5 or PR8 stimulation.

Supplementary Table 1. Genome constellation of recombinant H5N1 viruses for viral characteristic by mutation in HA.

Recombinant virus	HA[b]	NA	PB2	PB1	PA	NP	M	NS
22W_MVV	22W HA	22W NA	310-MVV[a]	PR8	PR8	PR8	PR8	PR8
21W _MVV	21W HA	22W NA	310-MVV	PR8	PR8	PR8	PR8	PR8
22W R90K	22W HA-R90K	22W NA	310-MVV	PR8	PR8	PR8	PR8	PR8
22W A93T	22W HA-A93T	22W NA	310-MVV	PR8	PR8	PR8	PR8	PR8
22W S125N	22W HA-S125N	22W NA	310-MVV	PR8	PR8	PR8	PR8	PR8
22W N193D	22W HA-N193D	22W NA	310-MVV	PR8	PR8	PR8	PR8	PR8
22W H110Y	22W HA-H110Y	22W NA	310-MVV	PR8	PR8	PR8	PR8	PR8
22W_KY	22W HA-R90K/H110Y	22W NA	310-MVV	PR8	PR8	PR8	PR8	PR8

[a] PB2 gene of A/chicken/Korea/01310/2001 (01310, H9N2) virus with I66M, I109V and I133V mutations.

[b] The HA cleavage site was attenuated from RERRRKR to ASGR.

Supplementary Table 2. Amino acid identity between the viruses used in serological assays or challenge studies and the vaccine strains.

a

Strain name	Percent nucleotide(amino acid) identities of Hemagglutinin (HA1)		
	**22W_KY**	**SNU 50-5**	**PR8**
**22W_KY** [a]		83.8% (84.6%)	57.5% (55.4%)
**SNU 50-5** [b]	83.8% (84.6%)		58.6% (56.6%)
**PR8** [c]	57.5% (55.4%)	58.6% (56.6%)	

b

Strain name	Percent nucleotide(amino acid) identities of Hemagglutinin (HA2)		
	**22W_KY**	**SNU 50-5**	**PR8**
**22W_KY** [a]		89.1% (95.95%)	71.75% (78.83%)
**SNU 50-5** [b]	89.1% (95.95%)		71.75% (79.73%)
**PR8** [c]	71.75% (78.83%)	71.75% (79.73%)	

c

Strain name	Percent nucleotide(amino acid) identities of Neuraminidase (NA)		
	**22W_KY**	**SNU 50-5**	**PR8**
**22W_KY** [a]		91.9% (97.2%)	78.6% (83.5%)
**SNU 50-5** [b]	91.9% (97.2%)		78.1% (84.0%)
**PR8** [c]	78.6% (83.5%)	78.1% (84.0%)	

[a] Vaccine strain

[b] A/Wild duck/Korea/SNU50-5/2009

[c] A/Puerto Rico/8/1934

Supplementary Table 3. Challenge viruses used for this study

Challenge virus	Hemagglutinin accession number	Neuraminidase accession number
A/Puerto Rico/8/34	AB671289.1	MZ310491.1
A/wild duck/korea/SNU50-5/2009	JX497768.1	JX497770.1
